# Supplementary material for: Systematic revision of Gatesona (Crassiclitellata, Lumbricidae), an endemic earthworm genus from the Massif Central (France)
Source: PLoS One. 2021 Sep 2;16(9):e0255978. doi: 10.1371/journal.pone.0255978 (PMC8412367; doi:10.1371/journal.pone.0255978)
Supplement: S1 File — (DOCX) [file pone.0255978.s001.docx]

| Species | Publication |
| --- | --- |
| *Allolobophora chaetophora* Bouche, 1972 | Domínguez et al. 2015; Pérez-Losada et al. 2015 |
| *Allolobophora chlorotica* Savigny*, 1826* | Domínguez et al. 2015; Pérez-Losada et al. 2015 |
| *Allolobophora dacica* Pop, 1938 | Domínguez et al. 2015; Pérez-Losada et al. 2015 |
| *Allolobophora dubiosa* Orley, 1881 | Domínguez et al. 2015; Pérez-Losada et al. 2015 |
| *Allolobophora mehadiensis* Rosa, 1895 | Domínguez et al. 2015; Pérez-Losada et al. 2015 |
| *Allolobophora moebii* Michaelsen, 1895 | Domínguez et al. 2015; Pérez-Losada et al. 2015 |
| *Allolobophora molleri* Rosa, 1889 | Pérez-Losada et al. 2009, 2015; Domínguez et al. 2015 |
| *Allolobophora robusta* Rosa, 1895 | Domínguez et al. 2015; Pérez-Losada et al. 2015 |
| *Aporrectodea caliginosa* Savigny, 1826 | Pérez-Losada et al. 2009, 2015; Domínguez et al. 2015 |
| *Aporrectodea georgii* Michaelsen, 1890 | Domínguez et al. 2015; Pérez-Losada et al. 2015 |
| *Aporrectodea jassyensis* Michaelsen, 1891 | Domínguez et al. 2015; Pérez-Losada et al. 2015 |
| *Aporrectodea limícola* Michaelsen, 1890 | Pérez-Losada et al. 2009, 2015; Domínguez et al. 2015 |
| *Aporrectodea longa* Ude, 1885 | Pérez-Losada et al. 2009, 2015; Domínguez et al. 2015 |
| *Aporrectodea nocturna* Evans, 1946 | Pérez-Losada et al. 2009, 2015; Domínguez et al. 2015 |
| *Aporrectodea rosea* Savigny, 1826 | Domínguez et al. 2015; Pérez-Losada et al. 2015 |
| *Aporrectodea trapezoides* Duges*, 1828* | Pérez-Losada et al. 2009, 2015; Domínguez et al. 2015 |
| *Aporrectodea tuberculata* Eisen, 1874 | Pérez-Losada et al. 2009, 2015; Domínguez et al. 2015 |
| *Carpetania elisae* Álvarez, 1977 | Domínguez et al. 2015; Pérez-Losada et al. 2015 |
| *Cataladrilus edwarsi* Qiu and Bouché, 1998 | Domínguez et al. 2015; Pérez-Losada et al. 2015 |
| *Cataladrilus montícola* Qiu and Bouché, 1998 | Domínguez et al. 2015; Pérez-Losada et al. 2015 |
| *Cernosvitovia dudichi* Zicsi and Sapkarev, 1982 | Domínguez et al. 2015; Pérez-Losada et al. 2015 |
| *Cernosvitovia rebeli* Rosa, 1897 | Domínguez et al. 2015; Pérez-Losada et al. 2015 |
| *Compostelandrilus bercianus* Dominguez, Aira, Porto, Diaz Cosin and Perez-Losada, 2017 | Domínguez et al. 2018 |
| *Compostelandrilus cyaneus* Briones and Diaz Cosin, 1993 | Domínguez et al. 2018 |
| *Compostelandrilus menciae* Dominguez, Aira, Porto, Diaz Cosin and Perez-Losada, 2017 | Domínguez et al. 2018 |
| *Criodrilus lacuum* Hoffmeister, 1845 | Domínguez et al. 2015; Pérez-Losada et al. 2015 |
| *Dendrobaena attemsi* Michaelsen, 1902 | Domínguez et al. 2015; Pérez-Losada et al. 2015 |
| *Dendrobaena cf. bíblica* Rosa, 1893 | Domínguez et al. 2015; Pérez-Losada et al. 2015 |
| *Dendrobaena illyrica Cognetti de Martiis, 1906* | Domínguez et al. 2015; Pérez-Losada et al. 2015 |
| *Dendrobaena jastrebensis* Mrsic and Sapkarev, 1987 | Domínguez et al. 2015; Pérez-Losada et al. 2015 |
| *Dendrobaena octaedra* Savigny, 1826 | Domínguez et al. 2015; Pérez-Losada et al. 2015 |
| *Dendrobaena pentheri* Rosa, 1905 | Domínguez et al. 2015; Pérez-Losada et al. 2015 |
| *Dendrobaena pygmaea* Friend, 1923 | Domínguez et al. 2015; Pérez-Losada et al. 2015 |
| *Dendrobaena veneta* Rosa, 1886 | Domínguez et al. 2015; Pérez-Losada et al. 2015 |
| *Diporodrilus pilosus* Bouche 1972 | Domínguez et al. 2015; Pérez-Losada et al. 2015 |
| *Eisenia andrei* Bouche, 1972 | Domínguez et al. 2015; Pérez-Losada et al. 2015 |
| *Eisenia balatonica* Pop, 1943 | Domínguez et al. 2015; Pérez-Losada et al. 2015 |
| *Eisenia fétida* Savigny, 1826 | Domínguez et al. 2015; Pérez-Losada et al. 2015 |
| *Eisenia lucens* Vaga, 1857 | Domínguez et al. 2015; Pérez-Losada et al. 2015 |
| *Eiseniella tetraedra* Savigny, 1826 | Domínguez et al. 2015; Pérez-Losada et al. 2015 |
| *Eiseniona albolineata* Diaz Cosin, Trigo and Mato, 1989 | Domínguez et al. 2015; Pérez-Losada et al. 2015 |
| *Eiseniona oliveirae* Rosa, 1894 | Domínguez et al. 2015; Pérez-Losada et al. 2015 |
| *Eisenoides carolinensis* Michaelsen, 1910 | Domínguez et al. 2015; Pérez-Losada et al. 2015 |
| *Eisenoides lonnbergi* Michaelsen, 1894 | Domínguez et al. 2015; Pérez-Losada et al. 2015 |
| *Eophila crodabepis* Paoletti et al., 2016 | Paoletti et al. 2016, De Sosa et al. 2019 |
| *Eophila gestroi* Cognetti de Martiis, 1905 | Paoletti et al. 2016, De Sosa et al. 2019 |
| *Eophila tellinii* Rosa, 1888 | Paoletti et al. 2016, De Sosa et al. 2019 |
| *Ethnodrilus zajonci* Bouche, 1972 | Jiménez et al. In press |
| *Galiciandrilus bertae* Diaz Cosin, Mato and Mascato, 1985 | Pérez-Losada et al. 2011, 2015; Domínguez et al. 2015 |
| *Galiciandrilus morenoe* Diaz Cosin, Calvin and Mato, 1985 | Pérez-Losada et al. 2011, 2015; Domínguez et al. 2015 |
| *Helodrilus cernosvitovianus* Zicsi, 1967 | Domínguez et al. 2015; Pérez-Losada et al. 2015 |
| *Helodrilus cortezi* Qiu and Bouche, 1998 | Domínguez et al. 2015; Pérez-Losada et al. 2015 |
| *Helodrilus patriarcalis* Rosa, 1893 | Domínguez et al. 2015; Pérez-Losada et al. 2015 |
| *Lumbricus castaneus* Savigny*, 1826* | Domínguez et al. 2015; Pérez-Losada et al. 2015 |
| *Lumbricus rubellus* Hoffmeister, 1843 | Domínguez et al. 2015; Pérez-Losada et al. 2015 |
| *Octodriloides boninoi* Omodeo, 1962 | Domínguez et al. 2015; Pérez-Losada et al. 2015 |
| *Octodrilus complanatus* Duges, 1928 | Domínguez et al. 2015; Pérez-Losada et al. 2015 |
| *Octodrilus exacystis* Rosa, 1896 | Domínguez et al. 2015; Pérez-Losada et al. 2015 |
| *Octodrilus gradinescui* Pop, 1938 | Domínguez et al. 2015; Pérez-Losada et al. 2015 |
| *Octodrilus pseudocomplanatus* Omodeo, 1962 | Domínguez et al. 2015; Pérez-Losada et al. 2015 |
| *Octodrilus transpadanus* Rosa, 1884 | Domínguez et al. 2015; Pérez-Losada et al. 2015 |
| *Octolasion cyaneum* Savigny, 1826 | Domínguez et al. 2015; Pérez-Losada et al. 2015 |
| *Octolasion lacteum* Orley, 1885 | Domínguez et al. 2015; Pérez-Losada et al. 2015 |
| *Octolasion montanum* Wessely, 1905 | Domínguez et al. 2015; Pérez-Losada et al. 2015 |
| *Panoniona leoni* Michaelsen, 1891 | Domínguez et al. 2015; Pérez-Losada et al. 2015 |
| *Postandrilus lavellei* Qiu and Bouche, 1998 | Pérez-Losada et al. 2011, 2015; Domínguez et al. 2015 |
| *Postandrilus majorcanus* Qiu and Bouche, 1998 | Pérez-Losada et al. 2011, 2015; Domínguez et al. 2015 |
| *Postandrilus medoakus* Qiu and Bouche, 1998 | Pérez-Losada et al. 2011, 2015; Domínguez et al. 2015 |
| *Postandrilus palmensis* Qiu and Bouche, 1998 | Pérez-Losada et al. 2011, 2015; Domínguez et al. 2015 |
| *Postandrilus sapkarevi* Qiu and Bouche, 1998 | Pérez-Losada et al. 2011, 2015; Domínguez et al. 2015 |
| *Proctodrilus antipai* Michaelsen, 1891 | Domínguez et al. 2015; Pérez-Losada et al. 2015 |
| *Prosellodrilus biauriculatus* Bouche, 1972 | Domínguez et al. 2015; Pérez-Losada et al. 2015 |
| *Prosellodrilus biserialis* Bouche, 1972 | Domínguez et al. 2015; Pérez-Losada et al. 2015 |
| *Prosellodrilus pyrenaicus* Cognetti, 1904 | Domínguez et al. 2015; Pérez-Losada et al. 2015 |
| *Scherotheca cf. gigas* Bouche, 1972 | Domínguez et al. 2015; Pérez-Losada et al. 2015 |
| *Scherotheca corsicana* Pop, 1947 | Domínguez et al. 2015; Pérez-Losada et al. 2015 |
| *Scherotheca savignyi* Bouche, 1972 | Domínguez et al. 2015; Pérez-Losada et al. 2015 |
| *Scherotheca* sp 1 | Pérez-Losada et al. 2009, 2015; Domínguez et al. 2015 |
| *Scherotheca* sp 2 | Pérez-Losada et al. 2009, 2015; Domínguez et al. 2015 |
| *Zophoscolex atlanticus* Bouche, 1972 | Jiménez et al. In press |
| *Zophoscolex graffi* Bouche, 1972 | Jiménez et al. In press |
| *Zophoscolex micellus* Bouche, 1972 | Jiménez et al. In press |
