# Supplementary figures and images for: Systematic revision of Gatesona (Crassiclitellata, Lumbricidae), an endemic earthworm genus from the Massif Central (France)
Source: PLoS One. 2021 Sep 2;16(9):e0255978. doi: 10.1371/journal.pone.0255978 (PMC8412367; doi:10.1371/journal.pone.0255978)

**
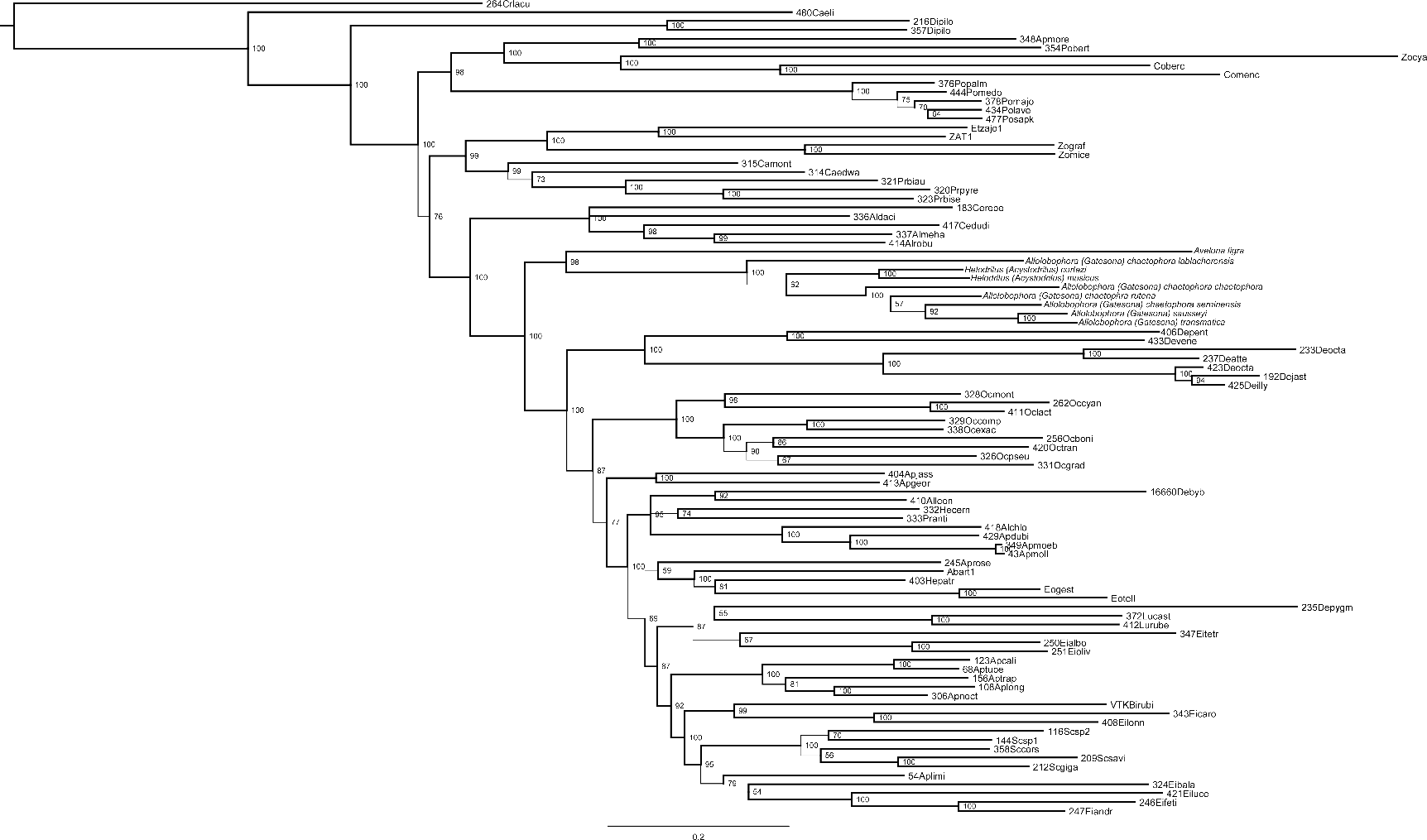
**

Supplement: S2 File — (DOCX) [file pone.0255978.s002.docx]
