# Supplementary material for: Systematic revision of Gatesona (Crassiclitellata, Lumbricidae), an endemic earthworm genus from the Massif Central (France)
Source: PLoS One. 2021 Sep 2;16(9):e0255978. doi: 10.1371/journal.pone.0255978 (PMC8412367; doi:10.1371/journal.pone.0255978)
Supplement: S3 File — (DOCX) [file pone.0255978.s003.docx]

|  | *Ap. caliginosa* | *Sc. savigny* | *D. octaedra* | *E. fetida* | *I. albolineatus* | *Ca. monticola* | *Pr. biauriculatus* | *Oc. complanatus* | *Pt. antipai* | *Ei. tetraedra* | *Po. majorcanus* | *Es. lonnbergi* | *Ot. lacteum* | *L. rubellus* | *All. chlorotica* | *Eo. tellinii* |
| --- | --- | --- | --- | --- | --- | --- | --- | --- | --- | --- | --- | --- | --- | --- | --- | --- |
| *Ap. caliginosa* |  |  |  |  |  |  |  |  |  |  |  |  |  |  |  |  |
| *Sc. savigny* | 8.6 |  |  |  |  |  |  |  |  |  |  |  |  |  |  |  |
| *D. octaedra* | 13.2 | 14.3 |  |  |  |  |  |  |  |  |  |  |  |  |  |  |
| *E. fetida* | 11.3 | 13.5 | 14.7 |  |  |  |  |  |  |  |  |  |  |  |  |  |
| *I. albolineatus* | 10.9 | 11.6 | 14.3 | 15.4 |  |  |  |  |  |  |  |  |  |  |  |  |
| *Ca. monticola* | 12.1 | 13.9 | 17.9 | 13.9 | 14.3 |  |  |  |  |  |  |  |  |  |  |  |
| *Pr. biauriculatus* | 15.5 | 16.6 | 17.6 | 17.7 | 15.5 | 13.9 |  |  |  |  |  |  |  |  |  |  |
| *Oc. complanatus* | 10.6 | 13.2 | 13.2 | 13.9 | 10.5 | 15.8 | 16.2 |  |  |  |  |  |  |  |  |  |
| *Pt. antipai* | 10.6 | 11.3 | 14.8 | 10.2 | 13.5 | 14.7 | 16.2 | 13.1 |  |  |  |  |  |  |  |  |
| *Ei. tetraedra* | 8.7 | 10.2 | 16.7 | 11.4 | 12.9 | 11.7 | 16.0 | 13.6 | 9.8 |  |  |  |  |  |  |  |
| *Po. majorcanus* | 14.0 | 15.1 | 16.7 | 15.5 | 15.1 | 15.5 | 20.1 | 18.9 | 15.5 | 15.2 |  |  |  |  |  |  |
| *Es. lonnbergi* | 9.5 | 12.9 | 17.2 | 13.6 | 14.4 | 15.2 | 17.5 | 14.0 | 12.5 | 14.1 | 15.0 |  |  |  |  |  |
| *Ot. lacteum* | 12.1 | 13.2 | 16.4 | 12.8 | 12.8 | 15.1 | 20.0 | 13.9 | 15.0 | 14.0 | 15.1 | 13.6 |  |  |  |  |
| *L. rubellus* | 9.8 | 13.6 | 16.8 | 12.1 | 12.1 | 13.3 | 17.8 | 12.8 | 13.6 | 11.4 | 14.8 | 12.9 | 13.2 |  |  |  |
| *All. chlorotica* | 9.8 | 9.8 | 10.9 | 10.6 | 10.6 | 11.7 | 14.7 | 12.8 | 10.1 | 10.6 | 12.5 | 12.5 | 12.0 | 12.4 |  |  |
| *Eo. tellinii* | 9.5 | 11.7 | 13.2 | 10.9 | 11.3 | 12.9 | 14.4 | 11.4 | 9.8 | 13.6 | 14.0 | 12.9 | 15.2 | 11.4 | 8.7 |  |
