# Supplementary material for: Systematic revision of Gatesona (Crassiclitellata, Lumbricidae), an endemic earthworm genus from the Massif Central (France)
Source: PLoS One. 2021 Sep 2;16(9):e0255978. doi: 10.1371/journal.pone.0255978 (PMC8412367; doi:10.1371/journal.pone.0255978)
Supplement: Attachment — Submitted filename: PONE-D-21-07434_reviewerSJ.pdf [file pone.0255978.s005.pdf]

PLOS ONE

Systematic revision of Gatesona (Crassiclitellata, Lumbricidae), an endemic earthworm genus from the Massif Central (France)

--Manuscript Draft--

**Manuscript Number:**	PONE-D-21-07434
**Article Type:**	Research Article
**Full Title:**	Systematic revision of Gatesona (Crassiclitellata, Lumbricidae), an endemic earthworm genus from the Massif Central (France)
**Short Title:**	Systematic revision of the earthworm genus Gatesona
**Corresponding Author:**	Daniel Fernández Marchán, Ph. D.<br>Universidade de Vigo Facultad de Ciencias<br>Vigo, SPAIN
**Keywords:**	Molecular phylogenetics, integrative taxonomy, species delimitation, earthworms, endemic
**Abstract:**	The Massif Central in France could potentially harbor numerous ancient endemic lineages owing to its long history of continuous geological stability. Several endemic earthworm species inhabit the area, with Allolobophora (Gatesona) chaetophora, Helodrilus (Acystodrilus) and Avelona ligra showing hints of a common evolutionary origin. However, the phylogenetic relationships and taxonomic status of the species remain to be studied through integrative molecular and morphological methods. To this end, eight species including most of the known species and subspecies of All. (Gatesona), Helodrilus (Acystodrilus) musicus, and Avelona ligra were sequenced for a set of five molecular markers. The species were grouped on the basis of the molecular findings in a phylogenetic framework. All. (Gatesona) was included within the same clade as Helodrilus (Acystodrilus) and Avelona, separated from Allolobophora sensu stricto, supporting its status as a good genus. Branch lengths and average pairwise genetic distances suggested the subspecies of All. (Gatesona) chaetophora examined should be considered species-level taxa. Thus, a generic diagnosis for Gatesona stat. nov. is provided, along with redescriptions of Gatesona rutena stat. nov. and Gatesona lablacherensis stat. nov. The study findings highlight the need for further sampling of earthworm diversity in the Massif Central (and Southern France), in addition to an increased focus on the Eastern European species of Helodrilus.
**Order of Authors:**	Daniel Fernández Marchán, Ph. D.<br>Sergio Jiménez<br>Jorge Domínguez
**Additional Information:**	
**Question**	**Response**
**Financial Disclosure**<br><br>Enter a financial disclosure statement that describes the sources of funding for the work included in this submission. Review the submission guidelines for detailed requirements. View published research articles from *PLOS ONE* for specific examples.<br><br>This statement is required for submission and **will appear in the published article** if the submission is accepted. Please make sure it is accurate.	This work was supported by a grant from the Systematic Research Fund and by Xunta de Galicia. Consellería de Cultura, Educación e Ordenación Universitaria. Secretaria Xeral de Universidades under grant ED431B 2019/038.<br>DFM was funded by a Juan de La Cierva-Formación grant (FJCI-2017-32895) from the Spanish Ministry of Sciences, Innovation and Universities.

**Unfunded studies**<br>Enter: *The author(s) received no specific funding for this work.*<br><br>**Funded studies**<br>Enter a statement with the following details:<br>• Initials of the authors who received each award<br>• Grant numbers awarded to each author<br>• The full name of each funder<br>• URL of each funder website<br>• Did the sponsors or funders play any role in the study design, data collection and analysis, decision to publish, or preparation of the manuscript?<br>• **NO** - Include this sentence at the end of your statement: *The funders had no role in study design, data collection and analysis, decision to publish, or preparation of the manuscript.*<br>• **YES** - Specify the role(s) played.<br><br>* typeset	
**Competing Interests**<br><br>Use the instructions below to enter a competing interest statement for this submission. On behalf of all authors, disclose any competing interests that could be perceived to bias this work—acknowledging all financial support and any other relevant financial or non-financial competing interests.<br><br>This statement is required for submission and **will appear in the published article** if the submission is accepted. Please make sure it is accurate and that any funding sources listed in your Funding Information later in the submission form are also declared in your Financial Disclosure statement.<br><br>View published research articles from *PLOS ONE* for specific examples.	The authors have declared that no competing interests exist.

Powered by Editorial Manager® and ProduXion Manager® from Aries Systems Corporation

**NO authors have competing interests**<br><br>Enter: *The authors have declared that no competing interests exist.*<br><br>**Authors with competing interests**<br><br>Enter competing interest details beginning with this statement:<br><br>*I have read the journal's policy and the authors of this manuscript have the following competing interests: [insert competing interests here]*<br><br>* typeset	
**Ethics Statement**<br><br>Enter an ethics statement for this submission. This statement is required if the study involved:<br><br>• Human participants<br>• Human specimens or tissue<br>• Vertebrate animals or cephalopods<br>• Vertebrate embryos or tissues<br>• Field research<br><br>Write "N/A" if the submission does not require an ethics statement.<br><br>General guidance is provided below. Consult the submission guidelines for detailed instructions. **Make sure that all information entered here is included in the Methods section of the manuscript.**	N/A

Powered by Editorial Manager® and ProduXion Manager® from Aries Systems Corporation

**Format for specific study types**<br><br>**Human Subject Research (involving human participants and/or tissue)**<br>• Give the name of the institutional review board or ethics committee that approved the study<br>• Include the approval number and/or a statement indicating approval of this research<br>• Indicate the form of consent obtained (written/oral) or the reason that consent was not obtained (e.g. the data were analyzed anonymously)<br><br>**Animal Research (involving vertebrate animals, embryos or tissues)**<br>• Provide the name of the Institutional Animal Care and Use Committee (IACUC) or other relevant ethics board that reviewed the study protocol, and indicate whether they approved this research or granted a formal waiver of ethical approval<br>• Include an approval number if one was obtained<br>• If the study involved *non-human primates*, add *additional details* about animal welfare and steps taken to ameliorate suffering<br>• If anesthesia, euthanasia, or any kind of animal sacrifice is part of the study, include briefly which substances and/or methods were applied<br><br>**Field Research**<br><br>Include the following details if this study involves the collection of plant, animal, or other materials from a natural setting:<br>• Field permit number<br>• Name of the institution or relevant body that granted permission	
**Data Availability**<br><br>Authors are required to make all data underlying the findings described fully available, without restriction, and from the time of publication. PLOS allows rare exceptions to address legal and ethical concerns. See the PLOS Data Policy and FAQ for detailed information.	Yes - all data are fully available without restriction

A Data Availability Statement describing where the data can be found is required at submission. Your answers to this question constitute the Data Availability Statement and **will be published in the article**, if accepted.<br><br>**Important:** Stating 'data available on request from the author' is not sufficient. If your data are only available upon request, select 'No' for the first question and explain your exceptional situation in the text box.<br><br>Do the authors confirm that all data underlying the findings described in their manuscript are fully available without restriction?	
**Describe where the data may be found in full sentences. If you are copying our sample text, replace any instances of XXX with the appropriate details.**<br><br>• If the data are **held or will be held in a public repository**, include URLs, accession numbers or DOIs. If this information will only be available after acceptance, indicate this by ticking the box below. For example: *All XXX files are available from the XXX database (accession number(s) XXX, XXX.)*.<br>• If the data are all contained **within the manuscript and/or Supporting Information files**, enter the following: *All relevant data are within the manuscript and its Supporting Information files.*<br>• If neither of these applies but you are able to provide **details of access elsewhere**, with or without limitations, please do so. For example:<br><br>*Data cannot be shared publicly because of [XXX]. Data are available from the XXX Institutional Data Access / Ethics Committee (contact via XXX) for researchers who meet the criteria for access to confidential data.*<br><br>*The data underlying the results presented in the study are available from (include the name of the third party*	All sequence files will be available from the Genbank database (accession number(s) XXX, XXX.).

and contact information or URL).<br>• This text is appropriate if the data are owned by a third party and authors do not have permission to share the data.<br><br>* typeset	
Additional data availability information:	Tick here if the URLs/accession numbers/DOIs will be available only after acceptance of the manuscript for publication so that we can ensure their inclusion before publication.

Powered by Editorial Manager® and ProduXion Manager® from Aries Systems Corporation

Manuscript

Click here to access/download;Manuscript;Manuscript_submission.docx

Full title:

Systematic revision of *Gatesona* (Crassiclitellata, Lumbricidae), an endemic earthworm genus from the Massif Central (France)

Short title: Systematic revision of the earthworm genus Gatesona

Daniel F. Marchán[1,#a,*], Sergio Jiménez[2], Jorge Domínguez[1]

[1] Grupo de Ecoloxía Animal (GEA), Universidade de Vigo, E-36310 Vigo, Spain

[2] Department of Biodiversity, Ecology and Evolution, Faculty of Biology, Universidad Complutense de Madrid, Madrid, Spain.

[#a] Current address: CEFE, UMR 5175, CNRS–Univ Montpellier–Univ Paul–Valéry–EPHE–SupAgro Montpellier–INRA–IRD, 1919 Route de Mende, 34293, Montpellier Cedex 5, France

*Corresponding author- Email address: danifermch@gmail.com

Abstract

The Massif Central in France could potentially harbor numerous ancient endemic lineages owing to its long history of continuous geological stability. Several endemic earthworm species inhabit the area, with *Allolobophora (Gatesona) chaetophora, Helodrilus (Acystodrilus)* and *Avelona ligra* showing hints of a common evolutionary origin. However, the phylogenetic relationships and taxonomic status of the species remain to be studied through integrative molecular and morphological methods. To this end, eight species including most of the known species and subspecies of *All. (Gatesona), Helodrilus (Acystodrilus) musicus,* and *Avelona ligra* were sequenced for a set of five molecular markers. The species were grouped on the basis of the molecular findings in a phylogenetic framework. *All. (Gatesona)* was included within the same clade as *Helodrilus (Acystodrilus)* and *Avelona,* separated from *Allolobophora* sensu stricto, supporting its status as a good genus. Branch lengths and average pairwise genetic distances suggested the subspecies of *All. (Gatesona) chaetophora* examined should be considered species-level taxa. Thus, a generic diagnosis for *Gatesona* stat. nov. is provided, along with redescriptions of *Gatesona rutena* stat. nov. and *Gatesona lablacherensis* stat. nov. The study findings highlight the need for further sampling of earthworm diversity in the Massif Central (and Southern France), in addition to an increased focus on the Eastern European species of *Helodrilus*.

Introduction

The Massif Central in France is an ancient massif which originated in the Variscan orogeny (Late Paleozoic) and has been an emerged continental realm through the Mesozoic and Cenozoic to the present [1]. Owing to this stability over geological time, the region is expected to harbour a large number of ancient endemic lineages (as observed for mite harvestmen in the Iberian Peninsula [2]. Earthworms, whose phylogeography and biogeography are known to reflect paleogeographic events [3], are a good example of this prediction. The Massif Central is inhabited by several endemic earthworm species from narrowly distributed genera: *Hemigastrodrilus monicae* Bouché 1970 (a basal lineage of Hormogastridae [4]), *Ethnodrilus aveli* Bouché 1972, *Ethnodrilus gatesi* Bouché 1972, *Zophoscolex graffi* Bouché 1972, *Zophoscolex micellus* Bouché 1972, *Allolobophora (Gatesona) chaetophora* Bouché 1972, *Helodrilus (Acystodrilus) cortezi* Qiu & Bouché 1998, *Helodrilus (Acystodrilus) musicus* Qiu & Bouché 1998, *Helodrilus (Acystodrilus) segalensis* Qiu & Bouché 1998 and *Avelona ligra* Bouché 1969.

Among the above-mentioned species, *All. (Ga.) chaetophora, Helodrilus (Acystodrilus)* and *Avelona ligra* may share a common evolutionary origin according to different sources of evidence. *All. (Ga.) chaetophora* and *He. (Ac.) cortezi* were grouped together in a relatively basal clade in the molecular phylogenetics analysis reported by Domínguez et al. [5]. This position is congruent with their morphological characteristics, as besides their nephridial bladder structure (sigmoid vs absent) both taxa are very similar. *Av. ligra* is also endemic to the (northeastern) Massif Central and morphologically similar to both taxa. The only molecular phylogenetics study including *Av. ligra* [6] found that it is closely related to *All. (Ga.) chaetophora*; however, the

sparse taxon sampling of Lumbricidae does not provide robust support for such a clade. In order to confirm a clade comprising these species, additional representatives of these taxa with a common molecular marker set must be examined.

In addition to the phylogenetic uncertainty, there remain some taxonomic questions regarding *All.* (*Ga.*) *chaetophora* Bouché (1972) described several subspecies within *All. (Ga.) chaetophora* with very restricted ranges and significant morphological differences from the type species. Bouché acknowledged that such morphological heterogeneity should be delimited in further studies (which he was unable to perform). Surprisingly, this type of study was performed in Qiu and Bouché [7] for other species complexes such as *Scherotheca* -resulting in the elevation of several subspecies to species status- but not for *All. (Ga.) chaetophora*. However, two closely related species, *All. (Ga.) sausseyi* and *All. (Ga.) transmatica*, were described and added to the subgenus *Gatesona*. The position of *Allolobophora chlorotica* (type species of *Allolobophora*) within a well-supported, independent clade from *All*. (*Ga*.) *chaetophora* [5] suggests that *Gatesona* could be erected as a proper genus. Molecular phylogenetic analysis of additional species (and subspecies) would contribute to a robust assessment of this hypothesis.

In this study, a systematic revision of *Allolobophora (Gatesona)* is proposed based on a molecular phylogenetic analysis including several species and subspecies. In addition, the phylogenetic link between *Allolobophora (Gatesona), Helodrilus (Acystodrilus)* and *Avelona ligra* was tested by the inclusion of the later in the same phylogenetic data set.

Materials and methods

Specimens, sampling and morphological description

The specimens described in this study were collected in a sampling survey carried out in Southeastern France and Catalonia between October and November 2019. The list of species and the locations where they were collected are shown in Table 1.

Earthworms were obtained by soil digging and hand-sorting, rinsed with water and fixed in 100% ethanol to enable further molecular analyses. Species classification and morphological diagnoses were conducted using the same set of external and internal morphological characters used by Qiu & Bouché [8][9]. The following main external morphological characters were considered: mean length, mean number of segments, mean weight, pigmentation, type of prostomium, setal arrangement, position of papillae, position of first dorsal pore, nephridial pore arrangement, position and development of male pores, position and development of female pores, position of spermathecal pores, position of clitellum and position of tubercula pubertatis.

The main internal anatomical characters considered were as follows: position of oesophageal hearts, position and morphology of calciferous glands, position of crop, position of gizzard, type of typhlosole, shape of nephridial bladders, number and position of seminal vesicles, and number and position of spermathecae.

Table 1. Species sampled and sequenced in this study, with their sampling location and coordinates.

Taxon	Location	Coordinates
Allolobophora (Gatesona) chaetophora lablacherensis	Lablachère, France	44.433, 4.22643
Allolobophora (Gatesona) chaetophora serninensis	Saint-Sernin-sur-Rance	43.87487, 2.60635
Allolobophora (Gatesona) chaetophora rutena	Rôdez, France	44.415, 2.03943
Allolobophora (Gatesona) sausseyi	Argelès-sur-Mer, France	42.52361, 3.04193
Allolobophora (Gatesona) transmatica	Agullana, Spain	42.39626, 2.84851
Helodrilus (Acystodrilus) musicus	Saint-Germain-de-Calberte	44.21667, 3.79999
Avelona ligra	To be provided	To be provided
Allolobophora bartolii	Jouques, France	43.641, 5.63043

DNA isolation, sequencing and molecular analyses

Total genomic DNA was extracted from ventral integument samples of approximately 5 x 5 mm by using the DNeasy Blood & Tissue Kit (Qiagen). For 8 species, regions of the nuclear 28S rRNA and mitochondrial 16S rRNA, tRNAs Ala, Leu and Ser, NADH dehydrogenase (*ND1*) and *COI* were amplified by polymerase chain reaction (PCR), with the primers and conditions described in Pérez-Losada *et al.* [10][11]. PCR products were purified and sequenced by the C.A.C.T.I Genomics service (University of Vigo).

DNA sequences obtained in this study are available in Genbank, under accession numbers XXXX-YYYYY.

Sequences reported by Domínguez *et al*. [5][12], Pérez-Losada *et al*. [10][11][13], Paoletti *et al*. [14] and de Sosa *et al*. [15], including representatives of most of the Lumbricidae genera and two members of the closest families (Hormogastridae and Criodrilidae), were downloaded from Genbank and used as a reference data set. The species included are listed in Supporting Information S1.

Sequences were aligned with MAFFT v.7 [16] with default settings and concatenated with BioEdit [17], resulting in a matrix of 3,201 bp. The best fitting evolutionary model for each partition was selected with jModelTest v. 2.1.3 [18] by applying the Akaike information criterion (AIC [19]), and Bayesian information criterion (BIC [20]). GTR + I + G was selected as the best-fitting evolutionary model for COI, 28S and ND1, HKY+I+G was selected for 16S and HKY+G was selected for tRNAs.

Maximum Likelihood analysis was performed with RaxML-NG [21] as implemented in the CIPRES Science Gateway V. 3.3 [22], with 10 random starting trees and estimating the support for the resulting topologies with 1,000 rapid bootstrap replicates. Bayesian Inference of the phylogeny was estimated with MRBAYES v.3.1.2 [23] as implemented in the CIPRES Science Gateway V. 3.3. Parameters were set to 50 million generations and sampled every 5,000th generation (10,000 trees). Two independent runs each with four chains were performed and 20% of the trees were discarded as burn-in. The remaining trees were combined and summarized on a 50% majority-rule consensus tree. Clade support values over 70% and 90% (for Bootstrap and Posterior probability respectively) were considered high (see [15][24]).

Uncorrected average pairwise distances between the studied species for the molecular markers COI and 16S were calculated in MEGA X [25] in order to support their status as separate species and to study genetic distances within and between genera.

Results and Discussion

Molecular phylogenetic analyses

Bayesian inference (Figure 1) and maximum likelihood inference phylogenetic trees recovered consistent topologies. *Avelona ligra* and representatives of *Helodrilus (Acystodrilus)* and *Allolobophora (Gatesona)* species formed a well-supported clade, isolated from species assigned to *Allolobophora* and *Helodrilus*. *Allolobophora (Gatesona)* and *Helodrilus (Acystodrilus)* appeared intermixed in a well-supported genus-level clade, hereafter referred to as *Gatesona*. Within *Gatesona*, representatives of the different subspecies of *All*. (*Ga.) chaetophora* were separated by long branches. In addition, *Ga. sausseyi* and *Ga. transmatica* were nested within a clade including *All*. (*Ga.) chaetophora* and its different subspecies.

Figure 1. Detail of the phylogenetic tree obtained by Bayesian phylogenetic analysis of the concatenated sequence of molecular markers *COI*–16S-tRNAs–*ND1*–28S (see full tree in Supporting Information S2). *Allolobophora (Gatesona)* representatives are shaded in blue and *Helodrilus (Acystodrilus)* representatives are shaded in green. Representatives of the main Lumbricidae genera were collapsed and are shown in boxes. Posterior probability support values are shown besides the corresponding nodes.

COI and 16s uncorrected average pairwise genetic (UAPG) distances between the species of the genera *Avelona, Gatesona* (comprising *All. (Gatesona*) and *He (Acystodrilus*)) and *Helodrilus (Helodrilus)* included in this study are shown in Table 2. UAPG distances between representatives of *Avelona*, *Gatesona* and *Helodrilus*

(Helodrilus) were high, ranging from 15.2% to 21% for COI and from 11.0% to 17.5% for 16s. UAPG distances within the genus-level clades were lower, with values corresponding to *Gatesona* (8.1%-17.3% COI, 4.1%-13.2% 16s) being generally lower than distances within *Helodrilus (Helodrilus)* (17% COI, 11.9% 16s). UAPG distances between the different species and subspecies of *Gatesona* were high (see above) except for *All. (Ga.) sausseyi- All. (Ga.) transmatica* (8.1% COI, 4.1% 16s).

Table 2. Uncorrected average pairwise genetic (UAPG) distances between species of the genera *Avelona, Helodrilus (Acystodrilus), Gatesona* and *Helodrilus* (*Helodrilus*) included in this study for the molecular markers COI (below the diagonal) and 16S (above the diagonal). Red shading indicates higher values and green shading indicates lower values. The COI UAPG distance between *All.* (*Ga.) rutena* and *He.* (*Ac.) musicus* is not displayed as it was unusually low.

	Av. ligra	*He. (Ac.) cortezi*	*He. (Ac.) musicus*	*All. (Ga.) chaetophora*	*All. (Ga.) chaetophora lab.*	*All. (Ga.) chaetophora ser.*	*All. (Ga.) chaetophora rutena*	*All. (Ga.) sausseyi*	*All. (Ga.) transmatica*	*He. cernosvitovianus*	*He. patriarchalis*
Av. ligra		14.3	16.9	16.6	13.5	12.4	13.5	14.6	15.7	13.3	13.1
He. (Ac.) cortezi	19.6		7.9	11.2	10.2	11.7	10.6	10.5	10.2	14.0	15.0
He. (Ac.) musicus	19.6	10.6		12.5	12.5	13.1	12.0	9.7	11.6	14.8	16.3
All. (Ga.) chaetophora	15.2	16.0	16.0		13.2	11.3	9.1	11.3	13.2	13.6	14.6
All. (Ga.) chaetophora lablacherensis	*	*	*	*		10.1	9.0	9.7	10.4	11.4	14.2
All. (Ga.) chaetophora serninensis	19.0	16.7	16.2	14.5	*		8.2	6.7	9.0	14.0	17.5
All. (Ga.) chaetophora rutena	17.8	9.1		13.6	*	14.6		7.5	7.9	11.0	13.5
All. (Ga.) sausseyi	18.3	17.3	16.5	14.4	*	14.5	14.1		4.1	14.8	17.1
All. (Ga.) transmatica	18.5	16.7	16.3	13.2	*	12.9	14.1	8.1		14.0	16.3
He. cernosvitovianus	20.5	19.0	18.8	17.5	*	19.5	17.3	19.8	19.1		11.9
He. patriarchalis	19.1	18.6	18.8	17.7	*	20.1	17.2	18.2	18.2	17.0	

Morphological analysis

The representatives of *Helodrilus* (*Acystodrilus) musicus* examined were wholly compatible with the available description [8] and did not differ in any morphological character.

External and internal morphological characters of the species and subspecies of *Allolobophora* (*Gatesona)* are shown in Table 3. Specimens were consistent with the available descriptions [9][26] except for the aspects detailed below.

Table 3. Main morphological characters of the species and subspecies of *Allolobophora* (*Gatesona)* under study. Species indicated with *: information from Bouché [26] and Qiu and Bouché [9]. In bold, morphological characters for which differences were observed relative to the original description [26].

Characters	*All. (Ga.) chaetophora**	*All. (Ga.) chaetophora lablacherensis*	*All. (Ga.) chaetophora serninensis**	*All. (Ga.) chaetophora rutena*	*All. (Ga.) sausseyi*	*All. (Ga.) transmatica*
Length	170-190 mm	88-100 mm		98-115 mm	86 mm	66 mm
N. segments	122-212	184-193		175-192	183	184
Weight	1.6-2 g	1.64-1.72g		2.16-3.47	1.25 g	1.03 g
Pigmentation	Pigmentary dots in cephalic segments	Pigmentary dots in cephalic (and tail) segments	-	Pigmentary dots in cephalic (and tail) segments	Pigmentary dots in cephalic (and tail) segments	Pigmentary dots in cephalic (and tail) segments
Prostomium	Epilobous	Epilobous	-	Epilobous	Epilobous	Epilobous
Setae	Closely paired	Closely paired	-	Closely paired	Closely paired	Closely paired
Setal arrangement	10:1.25:9.5:1:12.5	16:1:8.5:1:31	-	15:1:8.5:0.7:27.5	48.1:8:1:12	20:1:10:1:18
Papillae	(11, 14) 16 (17, 20, 21, 23, 26) 27-36.	13,14,16,17,19,23-33	-	12,16,17,24-36	18,23-33	11,17-18,23-33
First dorsal pore	(4/5)5/6	5/6	-	4/5	6/7	(6/7)7/8
Nephridial pores	En solfège	En solfège	-	En solfège	En solfège	En solfège
Male pores	1/2 15, *b*	1/2 15, *b*	-	1/2 15, *b*	1/2 15, *b*	1/2 15, *b*
Female pores	3/4 14, inconspicuous	3/4 14, inconspicuous	-	3/4 14, inconspicuous	1/2 14, b	1/2 14, b
Spermathecal pores	9/10, 10/11 simple in C	9/10, 10/11 simple in C	9/10, 10/11 simple in C	**9/10, 10/11 simple in C**	9/10, 10/11 simple in C	9/10, 10/11 simple in C

Clitellum	(26)27-36	25-32	25-32	26-35	24-33	25-33
Tubercula pubertatis	(26**)31-36	27-30, strongly developed	28-32	30-35	28-32	29-32
Oesophageal hearts	4-11	6-11	-	6-11	6-11	6-11
Calciferous glands	10-11(12)	10-12, dilated in 10	-	10-14, dilated in 10	10-14, diverticles in 10	10-12
Crop	15-16	15-16	15-16	15-16	15-16	15-16
Gizzard	17-18	17-18	17-18	17-18	17-18	17-18
Typhlosole	Bifid	Bifid	-	Bifid	Bifid	Bifid
Nephridial bladders	Sigmoid, proclinate	Sigmoid, proclinate	-	Sigmoid, proclinate	Sigmoid, proclinate	Sigmoid, proclinate
Seminal vesicles	9<10<11<12	**10,11,12**	9,10<11,12	9,10<11,12	9,10<11,12	9,11,12
Spermathecae	Simple, globular in 9,10	Simple, globular in 9,10	Simple, globular in 9,10	**Simple, globular in 9,10**	Simple, globular in 9,10	Simple, globular in 9,10

No mature individuals from the putative population of *All.* (*Ga.) serninensis* were collected, thus precluding comparison of their morphology with the description provided by Bouché [26].

Specimens of *All.* (*Ga.) lablacherensis* differed from the previous description in the absence of seminal vesicles in segment 9; however, these vesicles were expected to be very small (according to the previous information) and can sometimes be difficult to identify due to regression or incipient development.

Specimens of *All.* (*Ga.) rutena* differed from the previous description in the number and position of spermathecae (2 pairs in 9/10, 10/11 vs 3 pairs in 9/10, 10/11 and 11/12).

Systematic implications

The phylogenetic position of representatives of *Gatesona* as a well-supported clade unrelated to the generotype of *Allolobophora* (*All. chlorotica*) suggests it should be considered its own genus. The inclusion of *Helodrilus (Acystodrilus) cortezi* and *Helodrilus (Acystodrilus) musicus* within this clade suggest that they belong to the same

genus. The latter two species and the other representatives of *Gatesona* are very similar in most external and internal morphological characters, except for the absence of nephridial vesicles. This character appears in several unrelated lumbricid lineages (Erséus, Csudzi pers. comm.) and is thus homoplasic: the same is observed for the posterior displacement of the male pore, which has stopped being regarded as a key taxonomic character in lumbricid systematics [12][27]. Regression of a complex morphological structure is easier in evolutionary terms than the evolution of such structures [28], so it is likely that loss of nephridial bladders happened repeatedly associated with high moisture environments [29].

Other species placed within *Helodrilus (Acystodrilus*) by Qiu and Bouché [8] and not included in this work (*segalensis*, *putricola*, *tebra* and *rifensis*) could be expected to belong to the same genus-level clade as *Helodrilus (Acystodrilus*) *cortezi* and *Ac. musicus*, due to their morphological similarity and Western European distribution (Southern France and Morocco); however, molecular phylogenetics analyses of the species involved is necessary to confirm this hypothesis.

The name *Helodrilus* should be restricted to the clade containing *He. oculatus*, its generotype. However, *He. oculatus* was not included in this study, whereas only two Eastern European *Helodrilus (Helodrilus)* representatives among its 18 species (http://taxo.drilobase.org/) were included. Thus, a well-supported systematic revision of *Helodrilus* should include a more comprehensive sampling of both groups in a molecular phylogenetic framework.

Recovered as a sister clade to *Gatesona*, it could be argued that *Avelona* should also be included within the same genus. However, the branch lengths separating both taxa in the phylogenetic tree were indicative of separate genera. The 16s UAPG distances (which are less saturated at above-species level than COI distances) between *Avelona ligra* and

representatives of *Gatesona* were significantly higher than those found within *Gatesona*, and were within the range observed between well-established earthworm genera (8.6%-20.01%, average 13.4%; Supporting Information S2). In addition, *Avelona* possesses only two pairs of seminal vesicles in 11, 12 (vs 3 or 4 pairs in (9,10) 11, 12) and displays striking bioluminescence.

The phylogenetic relationships, branch lengths and UAPG distances between the different species and subspecies of *Gatesona* suggest that they should all be considered different species. The existing morphological differences between these taxa, of similar or higher magnitude than those used by Qiu and Bouché [9] to distinguish All. (*Ga.) sausseyi* and All. (*Ga.) transmatica*, support their status as proper species. This includes *All.* (*Ga.) chaetophora serninensis*: even though no mature specimens were ~~not~~ available for examination, the morphological differences with other *chaetophora* subspecies indicated by Bouché [26] together with the genetic differentiation observed are consistent with its recognition as a species.

All. (Ga.) argentatensis could not be sampled for this study, but following the same criteria it is very likely that it constitutes an independent species from *All.* (*Ga.) chaetophora*.

Proposed systematic changes

Phylum Annelida Lamarck, 1802

Class Oligochaeta Grube, 1850/Clitellata Michaelsen, 1919

Order Megadrili Benham, 1890/Haplotaxida Michaelsen, 1900

Family Lumbricidae Rafinesque-Schmaltz, 1815

Genus *Gatesona* Qiu and Bouché 1998 stat. nov.

Allolobophora (Gatesona) Qiu and Bouché 1998

Type species

Allolobophora chaetophora Bouché 1972

Gatesona chaetophora comb. nov. (Bouché 1972)

Gatesona chaetophora argentatensis comb.nov. (Bouché 1972)

Gatesona rutena comb.nov. stat. nov. (Bouché, 1972)

Gatesona lablacherensis comb.nov. (Bouché, 1972)

Gatesona serninensis comb.nov. stat. nov. (Bouché 1972)

Gatesona sausseyi comb. nov. (Qiu and Bouché 1998)

Gatesona transmatica comb. nov. (Qiu and Bouché 1998).

Gatesona cortezi comb. nov. (Qiu and Bouché 1998).

Gatesona musica comb. nov. (Qiu and Bouché 1998).

Diagnosis

Mid-sized (66 to 190 mm) Lumbricidae, with fewer than 230 segments. Mid-segment pigmentary dots in cephalic (sometimes tail) segments. Numerous chaetophores in the shape of genital papillae between segments 9 and the end of the clitellum. Prostomium epylobous. Closely paired setae. First dorsal pore in 4/5-7/8. Nephridial pores “en solfège” (irregularly distributed). Male pores in ½ 15 with developed porophore. Spermathecal pores simple, in 9/10, 10/11 (exceptionally 9/10, 10/11, 11/12). Clitellum starts between segments 23 to 27. Calciferous gland variable, in 10-12(14) usually

dilated or with diverticles in 10. Typhlosole bifid. Three to four pairs of seminal vesicles in (9, 10) 11, 12. Nephridial vesicles sigmoid, proclinate, sometimes absent (*Ga. cortezi, Ga. musica*).

Remarks

Gatesona chaetophora argentatensis (Bouché 1972) remains a subspecies of *Gatesona chaetophora* owing to failure to include representatives in the integrative systematic revision. However, it is to be expected that *Ga. chaetophora argentatensis* could represent an independent species.

No sexually mature specimens of *Gatesona serninensis comb.nov. stat. nov.* (Bouché 1972) were able to be examined in this study. Thus, this species was not redescribed as was the case for the other elevated species: until further specimens are collected and examined, the morphological characters shown in Table 3 (from Bouché, 1972) should be used for identification purposes.

Gatesona rutena comb.nov stat. nov.

Allolobophora chaetophora rutena Bouché 1972

Redescription

Diagnosis

Specimens of *Gatesona rutena* can be distinguished from other known species of *Gatesona* by the position of the clitellum in segments 26-35 and tubercula pubertatis in segments 30-35, calciferous glands in 10-14 and a higher mean body weight (Table 3).

Etymology

The name *rutena* refers to the toponym of Rodez, the region of France the species are known to inhabit.

Material examined

Holotype

FRANCE • Adult; Occitanie, Aveyron, Rodez; 10/11/2019; leg. Daniel F. Marchán; Locality 1: 44.416565 2.041011 Oak forest; deposited in UCMLT (voucher: 70257-1).

Paratypes

FRANCE • 6 specimens; same data as holotype. Deposited as follows: 6 specimens in UCMLT (vouchers: 70257-2,7)

Morphological description

External morphology

Body pigmentation very faint brown. White-beige with pigmentary dots scattered along the mid-segment line in the cephalic (sometimes tail) region in fixed specimens (Figure 2a,c).

Mean length 106.5 mm (98-115 mm, n = 2 adults); body cylindrical in cross-section; mean number of segments 183 (175-192, n = 2 adults; 192 segments in the holotype). Mean weight (fixed specimens): 2.82 g (2.16-3.47 g, n = 2 adults). Prostomium epilobous, closed. Longitudinal furrows in segments 1 and 2. First dorsal pore at intersegmental furrow 4/5. Nephridial pores "en solfège" (irregularly distributed). Spermathecal pores at intersegmental furrows 9/10 and 10/11 (plus 11/12 according to

Bouché 1972) in *c*. Male pores in segment 15, surrounded by a well-developed porophore. Female pores on the posterior part of segment 14, inconspicuous. Clitellum saddle-shaped in segments 26-35. Tubercula pubertatis in segments 30-35. Chaetae small and closely paired, with interchaetal ratio *aa*: 15, *ab*: 1, *bc*: 8.5, *cd*: 0.7, *dd*: 27.5 at segment 40. Chaetophores/genital papillae in segments 12, 16, 17, 27, 24-36.

Figure 2. External and internal morphology of *Gatesona rutena stat. nov*. (a, c) and *Gatesona lablacherensis stat. nov*. (b, d). a, b) Live specimens. c, d) Fixed specimens. The insets show a detailed view of the pigmentary dots characteristic of *Gatesona* species, and their nephridial bladders. Abbreviations: cl. = clitellum, m. p. = male pore, t. p.= tubercula pubertatis.

Internal anatomy

Septum 4/5 thickened, septa 5/6-8/9 strongly thickened. Hearts in segments 6-11, oesophageal. Calciferous glands in segments 10-14, dilated in segment 10. Crop in segments 15-16, gizzard in segments 17-18. Typhlosole bifid. Male sexual system holandric, testes and funnels (not enclosed in testes sacs, but with sperm present) located ventrally in segments 10 and 11. Four pairs of reniform seminal vesicles in segments 9, 10, 11 and 12, with the latter two pairs being larger. Ovaries and female funnels in segment 13, ovarian receptacles (ovisacs) in segment 14. Two pairs of globular spermathecae in segments 9 and 10 (plus 11 according to Bouché 1972). Nephridial bladders sigmoid, proclinate in anterior and posterior segments (Fig. 2c).

Distribution and ecology: *Gatesona rutena* is known from the region of Rodez, in the western Massif Central, France. This species was found in an oak forest, and it thus may prefer natural habitats.

Figure 2

Gatesona lablacherensis comb.nov stat. nov.

Allolobophora chaetophora lablacherensis Bouché 1972

Redescription

Diagnosis

Specimens of *Gatesona lablacherensis* can be distinguished from other known species of *Gatesona* by the position of the first dorsal pore in 4/5, clitellum in segments 25-32 and tubercula pubertatis in segments 27-30, as well as by possessing three pairs of seminal vesicles (in segments 10, 11 and 12) rather than four (Table 3).

Etymology

The name *lablacherensis* refer to Lablachère, the region of France the species ~~are~~ known to inhabit.

Holotype

FRANCE • Adult; Rhône-Alps, Ardèche, Lablachère; 6/11/2019; leg. Daniel F. Marchán; Locality 1: 44.433, 4.22643 Oak forest over rocky outcrops; deposited in UCMLT (voucher: 70256-1).

Paratypes

FRANCE • 11 specimens; same data as holotype. Deposited as follows: 11 specimens in UCMLT (vouchers: 70256-2,12)

Morphological description

External morphology

Body pigmentation absent. White-beige with pigmentary dots scattered along the mid-segment line in the cephalic region in fixed specimens (Figure 2b,d).

Mean length 94 mm (88-100 mm, n = 2 adults); body cylindrical in cross-section; mean number of segments 189 (184-193, n = 2 adults; 193 segments in the holotype). Mean weight (fixed specimens): 1.68 g (1.64-1.72 g, n = 2 adults). Prostomium epilobous, closed. Longitudinal furrows in segments 1 and 2. First dorsal pore at intersegmental furrow 5/6. Nephridial pores "en solfège" (irregularly distributed). Spermathecal pores at intersegmental furrows 9/10 and 10/11 in *c*. Male pores in segment 15, surrounded by a well-developed porophore. Female pores on the posterior part of segment 14, inconspicuous. Clitellum saddle-shaped in segments 25-32. Tubercula pubertatis in segments 27-30 strongly developed as downward projections of the body wall. Chaetae small and closely paired, with interchaetal ratio *aa*: 16, *ab*: 1, *bc*: 8.5, *cd*: 1, *dd*: 31 at segment 40. Chaetophores/genital papillae in segments 13, 14, 16, 17, 19, 23-33.

Internal anatomy

Septum 4/5 thickened, septa 5/6-8/9 strongly thickened. Hearts in segments 6-11, oesophageal. Calciferous glands in segments 10-12, dilated in segment 10. Crop in segments 15-16, gizzard in segments 17-18. Typhlosole bifid. Male sexual system holandric, testes and funnels (not enclosed in testes sacs, but with sperm present) located ventrally in segments 10 and 11. Three pairs of reniform seminal vesicles in segments 10, 11 and 12, with the latter two pairs being larger. Ovaries and female funnels in segment 13, ovarian receptacles (ovisacs) in segment 14. Two pairs of globular spermathecae in segments 9 and 10. Nephridial bladders sac-like or J shaped in the first segments, which gradually change to sigmoid, proclinate (Fig. 2d).

Distribution and ecology: *Gatesona lablacherensis* is known from the region of Lablachère, in the eastern Massif Central, France. This species has been found in an oak forest, and thus may prefer natural habitats.

Conclusions

Integrative systematics supported the status of the previously described subgenus *Allolobophora (Gatesona)* as a genus, including some representatives of *Helodrilus* (*Acystodrilus)*. Additionally, a clade comprising *Gatesona* and *Avelona* was recovered by molecular phylogenetics, an early branching clade with most of its species being endemic to the Massif Central. Morphological and molecular evidence indicated most of the previously described subspecies of *Gatesona chaetophora* should actually be regarded as proper species. The presence of this diverse assemblage of endemic earthworms, with several taxa remaining to be studied by molecular methods, highlights the interest of this region of Southern France for further sampling effort across their range.

The phylogenetic distinctness of representatives of *Helodrilus (Helodrilus)* and *Helodrilus* (*Acystodrilus)* indicate that a more comprehensive sampling of species (including *He. oculatus*) is required in order to establish a solid systematic revision of the elusive genus.

Acknowledgements

We are indebted to Marcel Koken who kindly provided the specimens of *Avelona ligra*.

This work was supported by a grant from the Systematic Research Fund and by Xunta de Galicia. Consellería de Cultura, Educación e Ordenación Universitaria. Secretaria Xeral de Universidades under grant ED431B 2019/038.

DFM was funded by a Juan de La Cierva-Formación grant (FJCI-2017-32895) from the Spanish Ministry of Sciences, Innovation and Universities.

References

[1] Peyaud JB, Barbarand J, Carter A, Pagel M. Mid-Cretaceous uplift and erosion on the northern margin of the Ligurian Tethys deduced from thermal history reconstruction. Int J Earth Sci. 2005; 94(3), 462-474.

[2] Murienne J, Giribet G. The Iberian Peninsula: ancient history of a hotspot of mite harvestmen (Arachnida, Opiliones, Cyphophthalmi, Sironidae) diversity. Zool J Linnean Soc. 2009; 156, 785–800.

[3] Fernández R, Almodóvar A, Novo M, Gutiérrez M, Díaz Cosín DJ. Earthworms, good indicators for palaeogeographical studies? Testing the genetic structure and demographic history in the peregrine earthworm *Aporrectodea trapezoides* (Dugès, 1828) in southern Europe. Soil Biol Biochem. 2013; 58, 127-135.

[4] Novo M, Fernández R, Andrade SC, Marchán DF, Cunha L et al. Phylogenomic analyses of a Mediterranean earthworm family (Annelida: Hormogastridae). Mol Phylogenetics Evol. 2016; 94, 473-478.

[5] Domínguez J, Aira M, Breinholt JW, Stojanovic M, James SW, Pérez-Losada M. Underground evolution: new roots for the old tree of lumbricid earthworms. Mol Phylogenetics Evol. 2015; 83, 7-19.

[6] Anderson FE, Williams BW, Horn KM, Erséus C, Halanych KM, Santos SR et al. Phylogenomic analyses of Crassiclitellata support major Northern and Southern Hemisphere clades and a Pangaean origin for earthworms. BMC Evol Biol. 2017; 17(1), 123.

[7] Qiu JP, Bouché M. Révision morphologique, chorologique et taxonomique du genre Scherotheca Bouché, 1972 (Oligochaeta: Lumbricidae). Doc. Pédozool. Integrol. 1998; 4, 117-139.

[8] Qiu JP, Bouché M. Une nouvelle évaluation du genere Helodrilus (sensu Zicsi, 1985) (Oligochaeta: Lumbricidae). Doc. Pédozool. Integrol. 1998; 4, 8-16.

[9] Qiu JP, Bouché M. Le genre Allolobophora Eisen, 1874 (Oligochaeta Lumbricidae), ses avatars et sa définition moderne. Doc. Pédozool. Integrol. 1998; 4, 86-97.

[10] Pérez-Losada M., Ricoy M, Marshall JC, Domínguez J. Phylogenetic assessment of the earthworm *Aporrectodea caliginosa* species complex (Oligochaeta: Lumbricidae) based on mitochondrial and nuclear DNA sequences. Mol Phylogenetics Evol. 2009; 52 (2), 293-302.

[11] Pérez-Losada M, Breinholt JW, Aira M, Domínguez J. An updated multilocus phylogeny of the Lumbricidae (Annelida: Clitellata: Oligochaeta) earthworms. J Phylogenetics Evol Biol. 2015; 3 (1).

[12] Domínguez J, Aira M, Porto PG, Díaz Cosín DJ, Pérez-Losada M. Multigene phylogeny reveals two new isolated and relic earthworm genera (Oligochaeta: Lumbricidae). Zool J Linnean Soc. 2018; 182(2), 258-274.

[13] Pérez-Losada M, Breinholt JW, Porto PG, Aira M, Domínguez J. An earthworm riddle: systematics and phylogeography of the Spanish lumbricid *Postandrilus*. PLoS One. 2011; 6 (11).

[14] Paoletti MG, Blakemore RJ, Csuzdi C, Dorigo L, Dreon AL, Gavinelli F. et al. Barcoding *Eophila crodabepis* sp. nov. (Annelida, Oligochaeta, Lumbricidae), a large stripy earthworm from alpine foothills of northeastern Italy similar to *Eophila tellinii* (Rosa, 1888). PloS One. 2016 ; 11 (3).

[15] de Sosa I, Díaz Cosín DJ, Csuzdi C, Paoletti MG, Marchán DF. Placing *Eophila tellinii* (Oligochaeta, Lumbricidae) in a molecular phylogenetic context advances the century-old controversy around the problematic genus. Eur J Soil Biol. 2019; 94.

[16] Katoh K, Standley D.M. MAFFT multiple sequence alignment software version 7: improvements in performance and usability. Mol Biol Evol. 2013; 30 (4), 772-780.

[17] Hall TA. BioEdit: a user-friendly biological sequence alignment editor and analysis program for Windows 95/98/NT. Nucleic Acids Symposium Series. 1999; 41, 95-98.

[18] Darriba D, Taboada GL, Doallo R, Posada D. jModelTest 2: more models, new heuristics and parallel computing. Nat Methods. 2012; 9 (8), 772.

[19] Akaike H. Maximum likelihood identification of Gaussian autoregressive moving average models. Biometrika. 1973; 60 (2), 255-265.

[20] Schwarz G. Estimating the dimension of a model. Ann Stat. 1978; 6 (2), 461-464.

[21] Stamatakis A. RAxML Version 8: A tool for Phylogenetic Analysis and Post-Analysis of Large Phylogenies. Bioinformatics. 2014; 30 (9), 1312-1313.

[22] Miller MA., Pfeiffer W, Schwartz T. Creating the CIPRES Science Gateway for inference of large phylogenetic trees. Gateway Computing Environments Workshop (GCE), New Orleans, LA. 2010, pp. 1-8

[23] Ronquist F, Huelsenbeck JP. MrBayes 3: Bayesian phylogenetic inference under mixed models. Bioinformatics. 2003; 19 (12), 1572-1574.

[24] Marchán DF, Fernández R, de Sosa I, Sánchez N, Díaz Cosín DJ, Novo M. Integrative systematic revision of a Mediterranean earthworm family: Hormogastridae (Annelida, Oligochaeta). Invertebr Syst. 2018; 32 (3), 652-671.

[25] Kumar S, Stecher G, Li M, Knyaz C, Tamura K. MEGA X: molecular evolutionary genetics analysis across computing platforms. Mol Biol Evol. 2018; 35 (6), 1547-1549.

[26] Bouché MB. Lombriciens de France: écologie et systématique (Vol. 72, No. 2). Paris: Institut National de la Recherche Agronomique. 1972

[27] Szederjesi T, Pop VV, Pavlícek, Márton O, Krízsik V, Csuzdi C. Integrated taxonomy reveals multiple species in the Dendrobaena byblica (Rosa, 1893) complex (Oligochaeta: Lumbricidae). Zool J Linnean Soc. 2018; 182(3), 500-516.

[28] Cresko WA, Amores A, Wilson C, Murphy J, Currey M, Phillips P. et al. Parallel genetic basis for repeated evolution of armor loss in Alaskan threespine stickleback populations. PNAS. 2004; 101(16), 6050-6055.

[29] Szederjesi T, Angyal D, Balázs G, Dány, L. Remarks on the earthworm genus Helodrilus Hoffmeister, 1845 with new epigean and subterranean records (Oligochaeta, Lumbricidae). Opusc Zool Budapest. 2014; 45(2), 181-188.

Supporting information captions

S1. Species included in the reference dataset

S2. Phylogenetic tree obtained by Bayesian phylogenetic analysis of the concatenated sequence of molecular markers *COI*–16S-tRNAs–*ND1*–28S.

S3. Uncorrected average pairwise (UAPG) distances for the marker 16S between representatives of different well-established genera of Lumbricidae.

Figure 1

Click here to access/download;Figure;Figure 1.tif

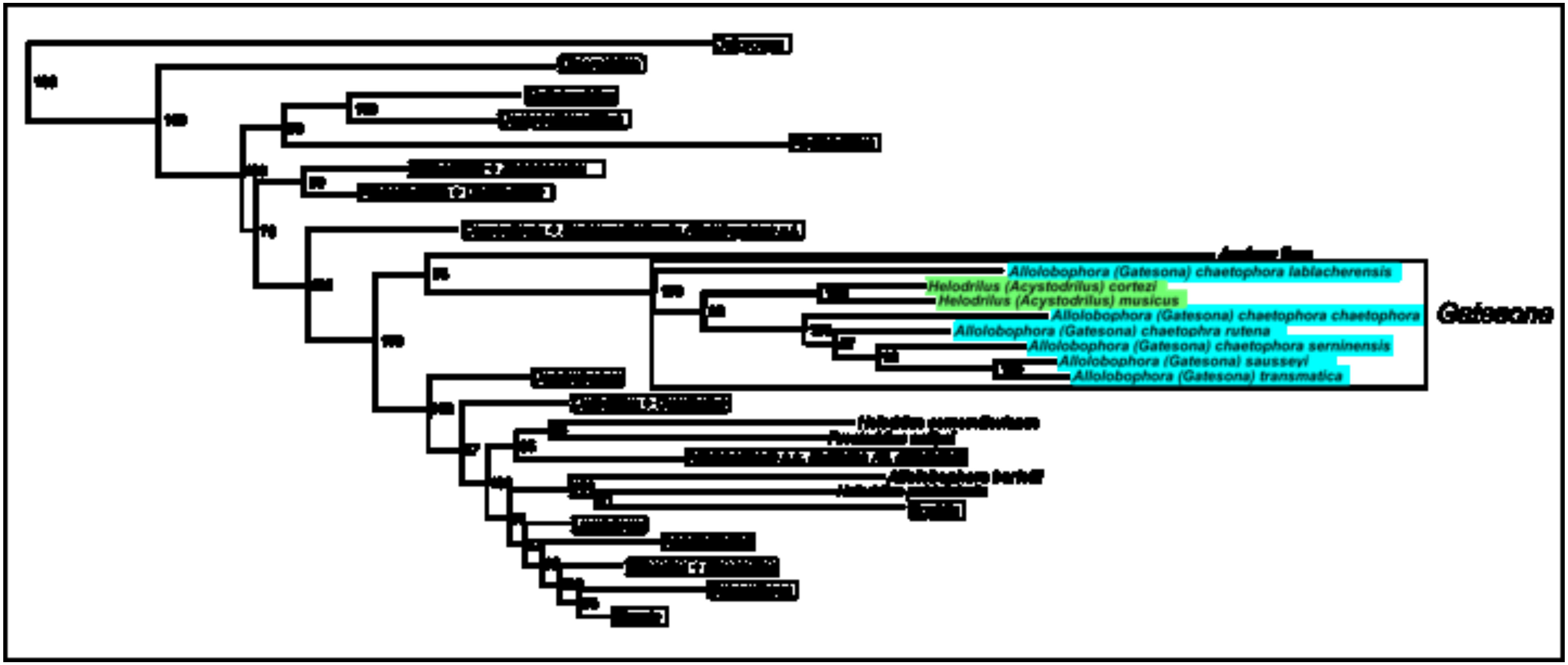

Figure 2

Click here to access/download;Figure;Figure 2.tif

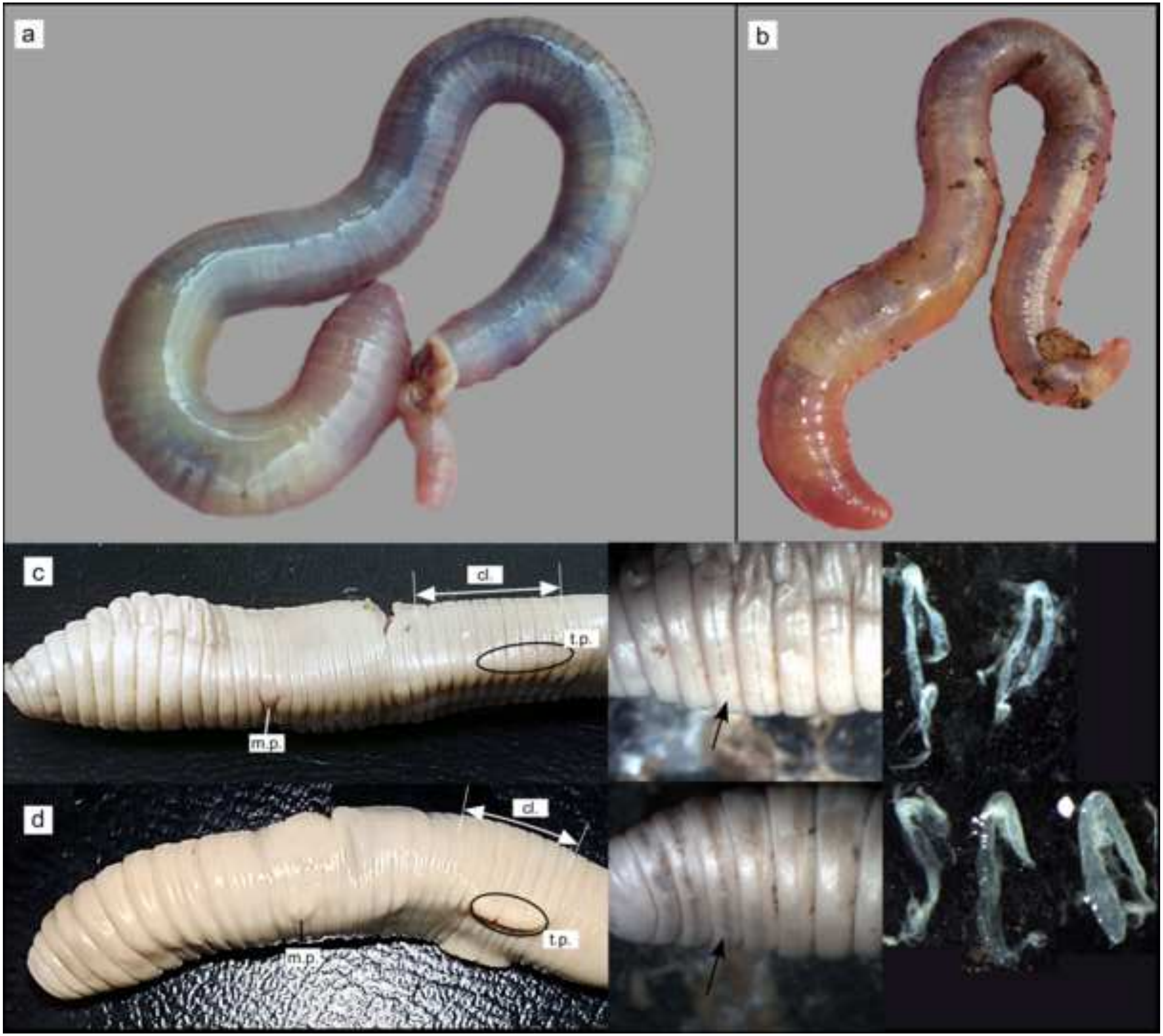

Supporting Information S1

Click here to access/download
Supporting Information
S1.docx

Click here to access/download
Supporting Information
S2.docx

Supporting Information S3

Click here to access/download
Supporting Information
S3.docx
