# Supplementary material for: Systematic revision of Gatesona (Crassiclitellata, Lumbricidae), an endemic earthworm genus from the Massif Central (France)
Source: PLoS One. 2021 Sep 2;16(9):e0255978. doi: 10.1371/journal.pone.0255978 (PMC8412367; doi:10.1371/journal.pone.0255978)
Supplement: Attachment — Submitted filename: PONE-D-21-07434_R1_reviewer 1 2nd round.pdf [file pone.0255978.s007.pdf]

PLOS ONE

Systematic revision of Gatesona (Crassiclitellata, Lumbricidae), an endemic earthworm genus from the Massif Central (France)

--Manuscript Draft--

**Manuscript Number:**	PONE-D-21-07434R1
**Article Type:**	Research Article
**Full Title:**	Systematic revision of Gatesona (Crassiclitellata, Lumbricidae), an endemic earthworm genus from the Massif Central (France)
**Short Title:**	Systematic revision of the earthworm genus Gatesona
**Corresponding Author:**	Daniel Fernández Marchán, Ph. D.<br>CEFE: Centre d'Ecologie Fonctionnelle et Evolutive<br>Montpellier, Occitanie FRANCE
**Keywords:**	Molecular phylogenetics, integrative taxonomy, species delimitation, earthworms, endemic
**Abstract:**	The Massif Central in France could potentially harbor numerous ancient endemic lineages owing to its long history of continuous geological stability. Several endemic earthworm species inhabit the area, with Allolobophora (Gatesona) chaetophora, Helodrilus (Acystodrilus) and Avelona ligra showing hints of a common evolutionary origin. However, the phylogenetic relationships and taxonomic status of the species remain to be studied through integrative molecular and morphological methods. To this end, eight species including most of the known species and subspecies of All. (Gatesona), Helodrilus (Acystodrilus) musicus, and Avelona ligra were sequenced for a set of five molecular markers. The species were grouped on the basis of the molecular findings in a phylogenetic framework. All. (Gatesona) was included within the same clade as Helodrilus (Acystodrilus) and Avelona, separated from Allolobophora sensu stricto, supporting its status as a good genus. Branch lengths and average pairwise genetic distances suggested the subspecies of All. (Gatesona) chaetophora examined should be considered species-level taxa. Thus, a generic diagnosis for Gatesona stat. nov. is provided, along with redescriptions of Gatesona chaetophora comb. nov., Gatesona rutena comb. nov. stat. nov., Gatesona lablacherensis comb. nov. stat. nov. and Gatesona serninensis comb. nov. stat. nov. The study findings highlight the need for further sampling of earthworm diversity in the Massif Central (and Southern France), in addition to an increased focus on the Eastern European species of Helodrilus .
**Order of Authors:**	Daniel Fernández Marchán, Ph. D.
	Sergio Jiménez
	Thibaud Decaëns
	Jorge Domínguez
**Response to Reviewers:**	Reviewer #1: Authors present a very interesting paper about the identity of several Lumbricidae species and subspecies that are placed in a new genus. These new arrangements are due to the use of an integrative approach (molecular genetics, phylogenetic analysis and morphological revisions). English is ok, as well as molecular and phylogenetic methods and results.<br><br>We want to thank the reviewer for his/her interest on our manuscript and his/her constructive suggestions in order to improve it. All of them have been considered and included.<br><br>There are, however, some modifications that should be done (mainly in regard to classical nomenclatural arrangements) before the paper could be accepted. The paper, thus, is ACCEPTED WITH MINOR CHANGES.<br><br>I directly wrote several comments and suggestions over the main text; following are some of my main points:

Powered by Editorial Manager® and ProduXion Manager® from Aries Systems Corporation

	1) In the title it is mentioned the word Crassiclitellata; in the classification of the new genus Gatesona, however, this word is not included. Thus, include in the supraorder classification this word with its hierarchical associated level.<br>We have included Crassiclitellata in the taxonomy section.<br><br>2) There are several mistakes in regard of the "types" of the two re-described species. Please attend comments of the text.<br>We have corrected the taxonomic errors regarding the types.<br><br>3) As authors do not reviewed types of these species, maybe the name (from an already described species) in fact does not correspond to the collected and molecular sequenced material. I strongly suggest contact Dr. Bouché in order to obtain in loan the types (or paratypes) to confirm that the morphology of Gatesona chaetophora rutena and Gatesona chaetophora lablacherensis really correspond to the species you collected. Even more because there is some variation that could be an indication of different morphological species (e.g. the number of spermathecae).<br>We have contacted Marcel Bouché and revised the holotypes for lablacherensis, rutena but also serninensis and chaetophora. Thanks to that, we can state with certainty that the (morpho-molecularly) studied material is morphologically compatible with the types of the original taxa. The morphological differences initially detected could be actually due to faulty/incomplete descriptions (in the end, it was done through tables and not through an orthodox text); when comparing with the types, the differences were non-existant or comprised within intra-individual variation (Gatesona rutena holotype possessed three spermathecae on one side but only two on the other).<br><br>4) In benefit of readers, please explain in the discussion why the genus was named as Gatesona and not Acystodrilus.<br><br>We have included a sentence explaining that point in the "Remarks" section of the Systematic changes to the genus Gatesona.<br><br>Reviewer #2: There are a few small corrections of grammar and content in the MS that I uploaded with this review. The revision of the taxonomy has to be modified to make it clear that G. chaetophora is the type species, and in particular, that G. chaetophora chaetophora is the type species. The subspecies G. c. argentatus could be tentatively excluded. The remaining species are "other species" under a heading.<br><br>We are thankful for the constructive revision and suggestions for improvement of our manuscript. As seen in the document with tracked changes, we have aknowledged and included all the suggested changes.<br>Most importantly, we contacted Marcel Bouché and revised the holotypes for lablacherensis, rutena but also serninensis and chaetophora. This allowed to check for morphological differences, actually ruling out most of them (holotypes were more consistent with our material tan suggested in the incomplete, informal descriptions provided in Bouché 1972). In addition, we sampled additional material of Gatesona serninensis, which allowed us to provide a formal redescription.<br>We have decided to mantain argentatensis within Gatesona chaetophora as it clearly belongs to the genus but we lack enough information to robustly elevate it to its own species.
**Additional Information:**	
**Question**	**Response**
**Financial Disclosure**<br><br>Enter a financial disclosure statement that describes the sources of funding for the work included in this submission. Review the submission guidelines for detailed requirements. View published research articles from *PLOS ONE* for specific examples.	This work was supported by a grant from the Systematic Research Fund and by Xunta de Galicia. Consellería de Cultura, Educación e Ordenación Universitaria. Secretaria Xeral de Universidades under grant ED431B 2019/038.<br>DFM was funded by a Juan de La Cierva-Formación grant (FJCI-2017-32895) from the Spanish Ministry of Sciences, Innovation and Universities.

Powered by Editorial Manager® and ProduXion Manager® from Aries Systems Corporation

This statement is required for submission and **will appear in the published article** if the submission is accepted. Please make sure it is accurate.<br><br>**Unfunded studies**<br>Enter: *The author(s) received no specific funding for this work.*<br><br>**Funded studies**<br>Enter a statement with the following details:<br>• Initials of the authors who received each award<br>• Grant numbers awarded to each author<br>• The full name of each funder<br>• URL of each funder website<br>• Did the sponsors or funders play any role in the study design, data collection and analysis, decision to publish, or preparation of the manuscript?<br>• **NO** - Include this sentence at the end of your statement: *The funders had no role in study design, data collection and analysis, decision to publish, or preparation of the manuscript.*<br>• **YES** - Specify the role(s) played.<br><br>* typeset	
**Competing Interests**<br><br>Use the instructions below to enter a competing interest statement for this submission. On behalf of all authors, disclose any competing interests that could be perceived to bias this work—acknowledging all financial support and any other relevant financial or non-financial competing interests.<br><br>This statement is required for submission and **will appear in the published article** if the submission is accepted. Please make sure it is accurate and that any funding sources listed in your Funding Information later in the submission form are also declared in your Financial Disclosure statement.<br><br>View published research articles from	The authors have declared that no competing interests exist.

PLOS ONE for specific examples.<br><br>**NO authors have competing interests**<br><br>Enter: *The authors have declared that no competing interests exist.*<br><br>**Authors with competing interests**<br><br>Enter competing interest details beginning with this statement:<br><br>*I have read the journal's policy and the authors of this manuscript have the following competing interests: [insert competing interests here]*<br><br>* typeset	
**Ethics Statement**<br><br>Enter an ethics statement for this submission. This statement is required if the study involved:<br><br>• Human participants<br>• Human specimens or tissue<br>• Vertebrate animals or cephalopods<br>• Vertebrate embryos or tissues<br>• Field research<br><br>Write "N/A" if the submission does not require an ethics statement.<br><br>General guidance is provided below. Consult the submission guidelines for detailed instructions. **Make sure that all information entered here is included in the Methods section of the manuscript.**	N/A

Powered by Editorial Manager® and ProduXion Manager® from Aries Systems Corporation

**Format for specific study types**<br><br>**Human Subject Research (involving human participants and/or tissue)**<br>• Give the name of the institutional review board or ethics committee that approved the study<br>• Include the approval number and/or a statement indicating approval of this research<br>• Indicate the form of consent obtained (written/oral) or the reason that consent was not obtained (e.g. the data were analyzed anonymously)<br><br>**Animal Research (involving vertebrate animals, embryos or tissues)**<br>• Provide the name of the Institutional Animal Care and Use Committee (IACUC) or other relevant ethics board that reviewed the study protocol, and indicate whether they approved this research or granted a formal waiver of ethical approval<br>• Include an approval number if one was obtained<br>• If the study involved *non-human primates*, add *additional details* about animal welfare and steps taken to ameliorate suffering<br>• If anesthesia, euthanasia, or any kind of animal sacrifice is part of the study, include briefly which substances and/or methods were applied<br><br>**Field Research**<br><br>Include the following details if this study involves the collection of plant, animal, or other materials from a natural setting:<br>• Field permit number<br>• Name of the institution or relevant body that granted permission	
**Data Availability**<br><br>Authors are required to make all data underlying the findings described fully available, without restriction, and from the time of publication. PLOS allows rare exceptions to address legal and ethical concerns. See the PLOS Data Policy and FAQ for detailed information.	Yes - all data are fully available without restriction

A Data Availability Statement describing where the data can be found is required at submission. Your answers to this question constitute the Data Availability Statement and **will be published in the article**, if accepted.<br><br>**Important:** Stating 'data available on request from the author' is not sufficient. If your data are only available upon request, select 'No' for the first question and explain your exceptional situation in the text box.<br><br>Do the authors confirm that all data underlying the findings described in their manuscript are fully available without restriction?	
**Describe where the data may be found in full sentences. If you are copying our sample text, replace any instances of XXX with the appropriate details.**<br><br>• If the data are **held or will be held in a public repository**, include URLs, accession numbers or DOIs. If this information will only be available after acceptance, indicate this by ticking the box below. For example: *All XXX files are available from the XXX database (accession number(s) XXX, XXX.*).<br>• If the data are all contained **within the manuscript and/or Supporting Information files**, enter the following: *All relevant data are within the manuscript and its Supporting Information files.*<br>• If neither of these applies but you are able to provide **details of access elsewhere**, with or without limitations, please do so. For example:<br><br>*Data cannot be shared publicly because of [XXX]. Data are available from the XXX Institutional Data Access / Ethics Committee (contact via XXX) for researchers who meet the criteria for access to confidential data.*<br><br>*The data underlying the results presented in the study are available from (include the name of the third party*	All sequence files are available in Genbank, under accession numbers MZ153163-MZ153169, MZ156573-MZ156588 and MZ235293-MZ235314.

and contact information or URL).<br>• This text is appropriate if the data are owned by a third party and authors do not have permission to share the data.<br><br>* typeset	
Additional data availability information:	Tick here if the URLs/accession numbers/DOIs will be available only after acceptance of the manuscript for publication so that we can ensure their inclusion before publication.

Powered by Editorial Manager® and ProduXion Manager® from Aries Systems Corporation

Manuscript Click here to access/download;Manuscript;Manuscript.docx

Full title:

Systematic revision of *Gatesona* (Crassiclitellata, Lumbricidae), an endemic earthworm genus from the Massif Central (France)

Short title: Systematic revision of the earthworm genus Gatesona

Daniel F. Marchán[1,*], Sergio Jiménez[2], Thibaud Decaëns[1], Jorge Domínguez[3]

[1] CEFE, UMR 5175, CNRS–Univ Montpellier–Univ Paul–Valéry–EPHE–SupAgro Montpellier–INRA–IRD, 1919 Route de Mende, 34293, Montpellier Cedex 5, France

[2] Department of Biodiversity, Ecology and Evolution, Faculty of Biology, Universidad Complutense de Madrid, Madrid, Spain.

[3]Grupo de Ecoloxía Animal (GEA), Universidade de Vigo, E-36310 Vigo, Spain

*Corresponding author- Email address: daniel.fernandez-marchan@umontpellier.fr

Abstract

The Massif Central in France could potentially harbor numerous ancient endemic lineages owing to its long history of continuous geological stability. Several endemic earthworm species inhabit the area, with *Allolobophora (Gatesona) chaetophora*, *Helodrilus (Acystodrilus)* and *Avelona ligra* showing hints of a common evolutionary origin. However, the phylogenetic relationships and taxonomic status of the species remain to be studied through integrative molecular and morphological methods. To this end, eight species including most of the known species and subspecies of *All. (Gatesona), Helodrilus (Acystodrilus) musicus,* and *Avelona ligra* were sequenced for a set of five molecular markers. The species were grouped on the basis of the molecular findings in a phylogenetic framework. *All. (Gatesona)* was included within the same clade as *Helodrilus (Acystodrilus)* and *Avelona,* separated from *Allolobophora* sensu stricto, supporting its status as a good genus. Branch lengths and average pairwise genetic distances suggested the subspecies of *All. (Gatesona) chaetophora* examined should be considered species-level taxa. Thus, a generic diagnosis for *Gatesona* stat. nov. is provided, along with redescriptions of *Gatesona chaetophora* comb. nov., *Gatesona rutena* comb. nov. stat. nov., *Gatesona lablacherensis* comb. nov. stat. nov. and *Gatesona serninensis* comb. nov. stat. nov. The study findings highlight the need for further sampling of earthworm diversity in the Massif Central (and Southern France), in addition to an increased focus on the Eastern European species of *Helodrilus*.

Introduction

The Massif Central in France is an ancient massif which originated in the Variscan orogeny (Late Paleozoic) and has been an emerged continental realm through the Mesozoic and Cenozoic to the present [1]. Owing to this stability over geological time, the region is expected to harbour a large number of ancient endemic lineages (as observed for mite harvestmen in the Iberian Peninsula [2]. Earthworms, whose phylogeography and biogeography are known to reflect paleogeographic events [3], are a good example of this prediction. The Massif Central is inhabited by several endemic earthworm species from narrowly distributed genera: *Hemigastrodrilus monicae* Bouché 1970 (a basal lineage of Hormogastridae [4]), *Ethnodrilus aveli* Bouché 1972, *Ethnodrilus gatesi* Bouché 1972, *Zophoscolex graffi* Bouché 1972, *Zophoscolex micellus* Bouché 1972, *Allolobophora (Gatesona) chaetophora* Bouché 1972, *Helodrilus (Acystodrilus) cortezi* Qiu & Bouché 1998, *Helodrilus (Acystodrilus) musicus* Qiu & Bouché 1998, *Helodrilus (Acystodrilus) segalensis* Qiu & Bouché 1998 and *Avelona ligra* Bouché 1969.

Among the above-mentioned species, *All. (Ga.) chaetophora, Helodrilus (Acystodrilus)* and *Avelona ligra* may share a common evolutionary origin according to different sources of evidence. *All. (Ga.) chaetophora* and *He. (Ac.) cortezi* were grouped together in a relatively basal clade in the molecular phylogenetics analysis reported by Domínguez et al. [5]. This position is congruent with their morphological characteristics, as excepting by their nephridial bladder structure (sigmoid vs absent) both taxa are very similar. *Av. ligra* is also endemic to the (northeastern) Massif Central and morphologically similar to both taxa. The only molecular phylogenetics study including *Av. ligra* [6] found that it is closely related to *All. (Ga.) chaetophora*;

however, the sparse taxon sampling of Lumbricidae does not provide robust support for such a clade. In order to confirm a clade comprising these species, additional representatives of these taxa with a common molecular marker set must be examined.

In addition to the phylogenetic uncertainty, there remain some taxonomic questions regarding *All.* (*Ga.*) *chaetophora*. Bouché (1972) described several subspecies within *All. (Ga.) chaetophora* with very restricted ranges and significant morphological differences from the type species. Bouché acknowledged that such morphological heterogeneity should be delimited in further studies (which he was unable to perform). Surprisingly, this type of study was performed in Qiu and Bouché [7] for other species complexes such as *Scherotheca* -resulting in the elevation of several subspecies to species status- but not for *All. (Ga.) chaetophora*. However, two closely related species, *All. (Ga.) sausseyi* Qiu & Bouché 1998 and *All. (Ga.) transmatica* Qiu & Bouché 1998, were described and added to the subgenus *Gatesona* [8]. The position of *Allolobophora chlorotica* (type species of *Allolobophora*) within a well-supported, independent clade from *All*. (*Ga*.) *chaetophora* [5] suggests that *Gatesona* could be erected as a proper genus. Molecular phylogenetic analysis of additional species (and subspecies) would contribute to a robust assessment of this hypothesis.

In this study, a systematic revision of *Allolobophora (Gatesona)* is proposed based on a molecular phylogenetic analysis including several species and subspecies. In addition, the phylogenetic link between *Allolobophora (Gatesona), Helodrilus (Acystodrilus)* and *Avelona ligra* was tested by the inclusion of the later in the same phylogenetic data set.

Materials and methods

Specimens, sampling and morphological description

The specimens described in this study were collected in a sampling survey carried out in Southeastern France and Catalonia between October and November 2019, with additional sampling in Spring 2021. The list of species and the locations where they were collected are shown in Table 1.

Earthworms were obtained by soil digging and hand-sorting, rinsed with water and fixed in 70% in order to obtain relaxed (as opposed to excessively retracted) specimens. They were subsequently transferred to 100% ethanol to enable further molecular analyses. Species classification and morphological diagnoses were conducted using the same set of external and internal morphological characters used by Qiu & Bouché [8][9]. The following main external morphological characters were considered: mean length, mean number of segments, mean weight, pigmentation, type of prostomium, setal arrangement, position of papillae, position of first dorsal pore, nephridial pore arrangement, position and development of male pores, position and development of female pores, position of spermathecal pores, position of clitellum and position of tubercula pubertatis.

The main internal anatomical characters considered were as follows: position of oesophageal hearts, position and morphology of calciferous glands, position of crop, position of gizzard, type of typhlosole, shape of nephridial bladders, number and position of seminal vesicles, and number and position of spermathecae.

Holotypes of *Allolobophora (Gatesona) chaetophora* and its different subspecies (see table 1), temporally available at the personal collection of Marcel Bouché, were studied and compared with the newly sampled material.

Table 1. Species sampled and sequenced in this study, with their sampling location and coordinates. *Individuals from this species were newly sampled for morphological study but previously existing sequences were used for the phylogenetic analyses.

Taxon	Location	Coordinates
*Allolobophora (Gatesona) chaetophora chaetophora**	L'Hospitalet du Larzac	43.956, 3.218433
Allolobophora (Gatesona) chaetophora lablacherensis Bouché 1972	Lablachère, France	44.433, 4.22643
Allolobophora (Gatesona) chaetophora serninensis Bouché 1972	Saint-Sernin-sur-Rance	43.87487, 2.60635
Allolobophora (Gatesona) chaetophora rutena Bouché 1972	Rôdez, France	44.415, 2.03943
Allolobophora (Gatesona) sausseyi	Argelès-sur-Mer, France	42.52361, 3.04193
Allolobophora (Gatesona) transmatica	Agullana, Spain	42.39626, 2.84851
Helodrilus (Acystodrilus) musicus	Saint-Germain-de-Calberte, France	44.21667, 3.79999
Avelona ligra	Jargeau, France	Not available
Allolobophora bartolii Bouché 1970	Jouques, France	43.641, 5.63043

DNA isolation, sequencing and molecular analyses

Total genomic DNA was extracted from ventral integument samples of approximately 5 x 5 mm by using the DNeasy Blood & Tissue Kit (Qiagen). For 8 species, regions of the nuclear 28S rRNA and mitochondrial 16S rRNA, tRNAs Ala, Leu and Ser, NADH dehydrogenase (*ND1*) and *COI* were amplified by polymerase chain reaction (PCR), with the primers and conditions described in Pérez-Losada *et al.* [10][11]. PCR products were purified and sequenced by the C.A.C.T.I Genomics service (University of Vigo).

DNA sequences obtained in this study are available in Genbank, under accession numbers MZ153163-MZ153169, MZ156573-MZ156588 and MZ235293-MZ235314.

Sequences reported by Domínguez *et al*. [5][12], Pérez-Losada *et al*. [10][11][13], Paoletti *et al*. [14] and de Sosa *et al*. [15], including representatives of most of the Lumbricidae genera and two members of the closest families (Hormogastridae and Criodrilidae), were downloaded from Genbank and used as a reference data set. The species included are listed in Supporting Information S1.

Sequences were aligned with MAFFT v.7 [16] with default settings and concatenated with BioEdit [17], resulting in a matrix of 3,201 bp. The best fitting evolutionary model for each partition was selected with jModelTest v. 2.1.3 [18] by applying the Akaike information criterion (AIC [19]), and Bayesian information criterion (BIC [20]). GTR + I + G was selected as the best-fitting evolutionary model for COI, 28S and ND1, HKY+I+G was selected for 16S and HKY+G was selected for tRNAs.

Maximum Likelihood analysis was performed with RaxML-NG [21] as implemented in the CIPRES Science Gateway V. 3.3 [22], with 10 random starting trees and estimating the support for the resulting topologies with 1,000 rapid bootstrap replicates. Bayesian Inference of the phylogeny was estimated with MRBAYES v.3.1.2 [23] as implemented in the CIPRES Science Gateway V. 3.3. Parameters were set to 50 million generations and sampled every 5,000th generation (10,000 trees). Two independent runs each with four chains were performed and 20% of the trees were discarded as burn-in. The remaining trees were combined and summarized on a 50% majority-rule consensus tree. Clade support values over 70% and 90% (for Bootstrap and Posterior probability respectively) were considered high (see [15][24]).

Uncorrected average pairwise distances between the studied species for the molecular markers COI and 16S were calculated in MEGA X [25] in order to support their status as separate species and to study genetic distances within and between genera.

Results and Discussion

Molecular phylogenetic analyses

Bayesian inference (Figure 1) and maximum likelihood inference phylogenetic trees recovered consistent topologies. *Avelona ligra* and representatives of *Helodrilus (Acystodrilus)* and *Allolobophora (Gatesona)* species formed a well-supported clade, isolated from species assigned to *Allolobophora* and *Helodrilus*. *Allolobophora (Gatesona)* and *Helodrilus (Acystodrilus)* appeared intermixed in a well-supported genus-level clade, hereafter referred to as *Gatesona*. Within *Gatesona*, representatives of the different subspecies of *All*. (*Ga.) chaetophora* were separated by long branches. In addition, *Ga. sausseyi* and *Ga. transmatica* were nested within a clade including *All*. (*Ga.) chaetophora* and its different subspecies.

Figure 1. Detail of the phylogenetic tree obtained by Bayesian phylogenetic analysis of the concatenated sequence of molecular markers *COI*–16S-tRNAs–*ND1*–28S (see full tree in Supporting Information S2). *Allolobophora (Gatesona)* representatives are shaded in blue and *Helodrilus (Acystodrilus)* representatives are shaded in green. Representatives of the main Lumbricidae genera were collapsed and are shown in boxes. Posterior probability support values are shown besides the corresponding nodes.

COI and 16s uncorrected average pairwise genetic (UAPG) distances between the species of the genera *Avelona, Gatesona* (comprising *All. (Gatesona*) and *He (Acystodrilus*)) and *Helodrilus (Helodrilus)* included in this study are shown in Table 2. UAPG distances between representatives of *Avelona, Gatesona* and *Helodrilus (Helodrilus)* were high, ranging from 15.2% to 21% for COI and from 11.0% to 17.5%

for 16s. UAPG distances within the genus-level clades were lower, with values corresponding to *Gatesona* (8.1%-17.3% COI, 4.1%-13.2% 16s) being generally lower than distances within *Helodrilus (Helodrilus)* (17% COI, 11.9% 16s). UAPG distances between the different species and subspecies of *Gatesona* were high (see above) except for *All. (Ga.) sausseyi- All. (Ga.) transmatica* (8.1% COI, 4.1% 16s).

Table 2. Uncorrected average pairwise genetic (UAPG) distances between species of the genera *Avelona, Helodrilus (Acystodrilus), Gatesona* and *Helodrilus* (*Helodrilus*) included in this study for the molecular markers COI (below the diagonal) and 16S (above the diagonal). Red shading indicates higher values and green shading indicates lower values. The COI distance between *All.* (*Ga.) rutena* and *He.* (*Ac.) musicus* is not displayed as it was unusually low. Asterisks indicate COI sequences were not available for the species.

	Av. ligra	*He. (Ac.) cortezi*	*He. (Ac.) musicus*	*All. (Ga.) chaetophora*	*All. (Ga.) chaetophora lab.*	*All. (Ga.) chaetophora ser.*	*All. (Ga.) chaetophora rutena*	*All. (Ga.) sausseyi*	*All. (Ga.) transmatica*	*He. cernosvitovianus*	*He. patriarchalis*
Av. ligra		14.3	16.9	16.6	13.5	12.4	13.5	14.6	15.7	13.3	13.1
He. (Ac.) cortezi	19.6		7.9	11.2	10.2	11.7	10.6	10.5	10.2	14.0	15.0
He. (Ac.) musicus	19.6	10.6		12.5	12.5	13.1	12.0	9.7	11.6	14.8	16.3
All. (Ga.) chaetophora	15.2	16.0	16.0		13.2	11.3	9.1	11.3	13.2	13.6	14.6
All. (Ga.) chaetophora lablacherensis	*	*	*	*		10.1	9.0	9.7	10.4	11.4	14.2
All. (Ga.) chaetophora serninensis	19.0	16.7	16.2	14.5	*		8.2	6.7	9.0	14.0	17.5
All. (Ga.) chaetophora rutena	17.8	9.1		13.6	*	14.6		7.5	7.9	11.0	13.5
All. (Ga.) sausseyi	18.3	17.3	16.5	14.4	*	14.5	14.1		4.1	14.8	17.1
All. (Ga.) transmatica	18.5	16.7	16.3	13.2	*	12.9	14.1	8.1		14.0	16.3
He. cernosvitovianus	20.5	19.0	18.8	17.5	*	19.5	17.3	19.8	19.1		11.9
He. patriarchalis	19.1	18.6	18.8	17.7	*	20.1	17.2	18.2	18.2	17.0	

Morphological analysis

External and internal morphological characters of the species and subspecies of *Allolobophora* (*Gatesona)* and related *Helodrilus (Acystodrilus)* species are shown in Table 3. Specimens were consistent with the holotypes and available descriptions [8][9][26] except for the aspects detailed below.

Specimens of *All*. (*Ga.) rutena* differed from the previous description in the number and position of spermathecal pores (2 pairs in 9/10, 10/11 vs 3 pairs in 9/10,10/11 and 11/12). However, the holotype of *All*. (*Ga.) rutena* showed 3 pairs of spermathecae on the right side but 2 pairs of spermathecae on the left side. Thus, this character appears to show variability even within the same individual.

Table 3. Main morphological characters of the species and subspecies of *Allolobophora* (*Gatesona)* and related *Helodrilus (Acystodrilus)* species under study. Species indicated with *: information from Qiu and Bouché [9]. In bold, morphological characters for which differences were observed relative to the original description [26].

Characters	*All. (Ga.) chaetophora*	*All. (Ga.) chaetophora lablacherensis*	*All. (Ga.) chaetophora serninensis*	*All. (Ga.) chaetophora rutena*	*All. (Ga.) sausseyi*	*All. (Ga.) transmatica*	*Helodrilus (Ac.) cortezi**	*Helodrilus (Ac.) musicus*
Length	68-85 mm	88-100 mm	70-99 mm	98-115 mm	86 mm	66 mm	152-192 mm	127 mm
N. segments	190	184-193	118-147	175-192	183	184	185-191	197
Weight	2.04-3.18 g	1.64-1.72g	1.085-1.675 g	2.16-3.47 g	1.25 g	1.03 g	1.10-1.36 g	1.42 g
Pigmentation	Pigmentary dots	Pigmentary dots	Pigmentary dots	Pigmentary dots	Pigmentary dots	Pigmentary dots	Pigmentary dots	**Pigmentary dots**
Setal arrangement	10:1.25:9.5:1:12.5	16:1:8.5:1:31	13.3:1:8.3:1:33	15:1:8.5:0.7:27.5	48:1:8:1:12	20:1:10:1:18	11.6:1.6:8.33:1:30	7:1:4:1:22
First dorsal pore	5/6	5/6	5/6	4/5	6/7	(6/7)7/8	4/5	4/5
Spermathecal pores	9/10, 10/11 simple in C	9/10, 10/11 simple in C	9/10, 10/11 simple in C	**9/10, 10/11 simple in C**	9/10, 10/11 simple in C	9/10, 10/11 simple in C	9/10, 10/11 simple in C	9/10, 10/11 simple in C
Clitellum	26-36	25-32	25-33	26-35	24-33	25-33	1/2 22-33	23-32
Tubercula pubertatis	(29)30-36	27-30, strongly developed	28-32(33)	30-35	28-32	29-32	28-30	27-1/2 31
Oesophageal hearts	5-11	6-11	6-12	6-11	6-11	6-11	6-11	6-11
Calciferous glands	10-12, dilated in 10	10-12, dilated in 10	10-12, diverticles in 10	10-14, dilated in 10	10-14, diverticles in 10	10-12	10-14	½ 10-14, diverticles in 10
Typhlosole	Bifid	Bifid	Bifid	Bifid	Bifid	Bifid	Bifid	Bifid
Nephridial bladders	Sigmoid, proclinate	Sigmoid, proclinate	Sigmoid, proclinate	Sigmoid, proclinate	Sigmoid, proclinate	Sigmoid, proclinate	Absent	Absent
Seminal vesicles	9<10<11,12	10,11,12	9,10<11,12	9,10<11,12	9,10<11,12	9,11,12	9,10<11,12	9,10<11,12
Spermathecae	Simple, globular in 9,10	Simple, globular in 9,10	Simple, globular in 9,10	**Simple, globular in 9,10**	Simple, globular in 9,10	Simple, globular in 9,10	Simple, globular in 9,10	Simple, globular in 9,10

Specimens of *Helodrilus (Ac.) musicus* did not show red pigmentation as stated by [9], but displayed a faint reddish hue instead.

Nephridial pores were defined by [8][9][26] as irregularly distributed; however, newly captured material showed aligned nephridial pores.

Systematic implications

The phylogenetic position of representatives of *Gatesona* as a well-supported clade unrelated to the generotype of *Allolobophora* (*All. chlorotica*) suggests it should be considered its own genus. The inclusion of *Helodrilus (Acystodrilus) cortezi* and *Helodrilus (Acystodrilus) musicus* within this clade suggest that they also belong to *Gatesona*. The latter two species and the other representatives of *Gatesona* are very similar in most external and internal morphological characters, except for the absence of nephridial vesicles. This character appears in several unrelated lumbricid lineages (Erséus, Csudzi pers. comm.) and is thus homoplasic: the same is observed for the posterior displacement of the male pore, which has stopped being regarded as a key taxonomic character in lumbricid systematics [12][27]. Regression of a complex morphological structure is easier in evolutionary terms than the evolution of such structures [28], so it is likely that loss of nephridial bladders happened repeatedly associated with high moisture environments [29].

Another species placed within *Helodrilus (Acystodrilus*) by Qiu and Bouché [9] and not included in this work*, Helodrilus (Acystodrilus*) *segalensis,* could be expected to belong to the same genus-level clade as *Helodrilus (Acystodrilus*) *cortezi* and *Ac. musicus*, due to their morphological similarity and geographic proximity (100-120 km); however, molecular phylogenetics analyses of this species is necessary to confirm this hypothesis. Furthermore, *Helodrilus putricola* and *tebra* have been found to belong to an unrelated

clade (personal communication) and *Helodrilus rifensis* is considered a synonym of *Eiseniella neapolitana* (Orley 1885) [30].

The name *Helodrilus* should be restricted to the clade containing *He. oculatus*, its generotype. However, *He. oculatus* was not included in this study, whereas only two Eastern European *Helodrilus (Helodrilus)* representatives (*Helodrilus patriarchalis* and *Helodrilus cernosvitovianus*) among its 18 species (http://taxo.drilobase.org/) were included. Thus, a well-supported systematic revision of *Helodrilus* should include a more comprehensive sampling of both groups in a molecular phylogenetic framework.

As *Avelona ligra* was recovered as a sister clade to *Gatesona*, it could be argued that it should also be included within the same genus. However, the branch lengths separating both taxa in the phylogenetic tree were indicative of separate genera. The 16s UAPG distances (which are less saturated at above-species level than COI distances) between *Avelona ligra* and representatives of *Gatesona* were significantly higher than those found within *Gatesona*, and were within the range observed between well-established earthworm genera (8.6%-20.01%, average 13.4%; Supporting Information S2). In addition, *Avelona ligra* (only known species within *Avelona*) possesses only two pairs of seminal vesicles in 11, 12 (vs 3 or 4 pairs in (9,10) 11, 12) and displays striking bioluminescence.

The phylogenetic relationships, branch lengths and UAPG distances between the different species and subspecies of *Gatesona* suggest that they should all be considered different species. The existing morphological differences between these taxa, of similar or higher magnitude than those used by Qiu and Bouché [8] to distinguish All. (*Ga.) sausseyi* and All. (*Ga.) transmatica*, support their status as proper species.

All. (Ga.) argentatensis could not be sampled for this study, but following the same criteria it is very likely that it constitutes an independent species from *All.* (*Ga.) chaetophora*.

Proposed systematic changes

Phylum Annelida Lamarck, 1802

Class Oligochaeta Grube, 1850/Clitellata Michaelsen, 1919

Order Crassiclitellata Jamieson, 1988

Family Lumbricidae Rafinesque-Schmaltz, 1815

Genus *Gatesona* Qiu and Bouché 1998 stat. nov.

Allolobophora (Gatesona) Qiu and Bouché 1998

Type species

Allolobophora chaetophora Bouché 1972

Included species

Gatesona chaetophora comb. nov. (Bouché 1972)

Gatesona chaetophora argentatensis comb.nov. (Bouché 1972)

Gatesona rutena comb.nov. stat. nov. (Bouché, 1972)

Gatesona lablacherensis comb.nov. (Bouché, 1972)

Gatesona serninensis comb.nov. stat. nov. (Bouché 1972)

Gatesona sausseyi comb. nov. (Qiu and Bouché 1998)

Gatesona transmatica comb. nov. (Qiu and Bouché 1998).

Gatesona cortezi comb. nov. (Qiu and Bouché 1998).

Gatesona musica comb. nov. (Qiu and Bouché 1998).

Diagnosis

Mid-sized (66 to 190 mm) Lumbricidae, with fewer than 230 segments. Mid-segment pigmentary dots in cephalic (sometimes tail) segments. Numerous chaetophores in the shape of genital papillae between segments 9 and the end of the clitellum. Prostomium epylobous. Closely paired setae. First dorsal pore in 4/5 (3 species), 5/6 (3 species), 6/7 (1 species), 7/8 (1 species). Nephridial pores aligned. Male pores in ½ 15 with developed porophore. Spermathecal pores simple, in 9/10, 10/11 (exceptionally 9/10, 10/11, 11/12). Clitellum starts between segments 23 to 27. Calciferous gland variable, in 10-12(14) usually dilated or with diverticles in 10. Typhlosole bifid. Three to four pairs of seminal vesicles in (9, 10) 11, 12. Nephridial vesicles sigmoid, proclinate, sometimes absent (*Ga. cortezi, Ga. musica*).

Remarks

Gatesona chaetophora argentatensis (Bouché 1972) remains a subspecies of *Gatesona chaetophora* owing to failure to include representatives in the integrative systematic revision. However, it is to be expected that *Ga. chaetophora argentatensis* could represent an independent species.

The name *Gatesona* was chosen over *Acystodrilus* for the genus encompassing species of both former subgenera for two main reasons: first, currently there are more species of the former *Allolobophora* (*Gatesona*) than of *Helodrilus* (*Acystodrilus*); second, the

name *Acystodrilus* (meaning "without vesicle") does not represent the more common character state within the redefined genus *Gatesona*.

***Gatesona chaetophora* comb.nov stat. nov.**

Allolobophora chaetophora chaetophora Bouché 1972

Redescription

Diagnosis

Specimens of *Gatesona chaetophora* can be distinguished from other known species of *Gatesona* by the position of the clitellum in segments 26-36 and tubercula pubertatis in segments (29)30-36 (Table 3).

Etymology

The name *chaetophora* refers to the multiple chaetophores (glandular structures surrounding genital chaetae) this species possess.

Material examined

Holotype

FRANCE • Adult; Occitanie, Aveyron, L'Hospitalet du Larzac; 11/03/1968; leg. Marcel Bouché; Locality 1: 43.956 3.218433 Prairie; deposited in the collection of the Muséum National d'Histoire Naturelle de Paris (MNHN) (voucher: 87/550/0711).

Additional material

FRANCE • 2 specimens, adults; Occitanie, Aveyron, L'Hospitalet du Larzac; 11/02/2021; leg. Daniel F. Marchán; Locality 1: 43.956, 3.218433 Prairie; deposited in the CEFE earthworm collection (voucher: DFM-0676 – DFM-0677).

Morphological description

External morphology

Body pigmentation faint brown. Beige to faint brown with pigmentary dots scattered along the mid-segment line in the cephalic region in fixed specimens (Figure 3a,e).

Mean length 76.5 mm (68-85 mm, n = 2 adults); body cylindrical in cross-section; mean number of segments 190 (n = 2 adults; 190 segments in the holotype). Mean weight (fixed specimens): 2.60 g (2.035-3.175 g, n = 2 adults). Prostomium epilobous, closed. Longitudinal furrows in segments 1 and 2. First dorsal pore at intersegmental furrow 45/6. Nephridial pores aligned in B close to b. Spermathecal pores at intersegmental furrows 9/10 and 10/11 in *c*. Male pores in segment 15, surrounded by a well-developed porophore. Female pores on the posterior part of segment 14, inconspicuous. Clitellum saddle-shaped in segments 26-36. Tubercula pubertatis in segments (29)30-36. Chaetae small and closely paired, with interchaetal ratio *aa*: 10, *ab*: 1.25, *bc*: 9.5, *cd*: 1, *dd*: 12.5 at segment 40. Chaetophores/genital papillae in segments 11, (14, 16, 17, 18) 20, 25-28, (29), 32-36 with those in 11, 20, 25-28 and 35,36 especially well developed and constant.

Figure 2. External morphology of living specimens of a) *Gatesona rutena* b) *Gatesona lablacherensis* c) *Gatesona serninensis*.

Figure 3. External and internal morphology of fixed specimens of a) *Gatesona chaetophora* b) *Gatesona rutena* c) *Gatesona lablacherensis* d) *Gatesona serninensis*. The insets show a detailed view of the pigmentary dots characteristic of *Gatesona* species (e, f, g, h), and their nephridial bladders (i, j, k, l). Abbreviations: cl. = clitellum, m. p. = male pore, t. p.= tubercula pubertatis.

Internal anatomy

Septa 5/6-9/10 strongly thickened. Hearts in segments 5-11, oesophageal. Calciferous glands in segments 10-12, dilated in segment 10. Crop in segments 15-16, gizzard in segments 17-18. Typhlosole bifid. Male sexual system holandric, testes and funnels (not enclosed in testes sacs, but with sperm present) located ventrally in segments 10 and 11. Four pairs of reniform seminal vesicles in segments 9, 10, 11 and 12, with the latter two pairs being larger. Ovaries and female funnels in segment 13, ovarian receptacles (ovisacs) in segment 14. Two pairs of globular spermathecae in segments 9 and 10. Nephridial bladders sigmoid, proclinate in anterior and posterior segments (Fig. 3i).

Distribution and ecology: *Gatesona chaetophora* is known from the region of Larzac in the southern Massif Central, France. This species was found at an altitude of 710 meters in a prairie on silty soil, with close to neutral pH (7.4), 2.83% organic matter and 0.308% nitrogen content [26].

Gatesona rutena comb.nov stat. nov.

Allolobophora chaetophora rutena Bouché 1972

Redescription

Diagnosis

Specimens of *Gatesona rutena* can be distinguished from other known species of *Gatesona* by the position of the first dorsal pore in 4/5, the position of the clitellum in segments 26-35 and tubercula pubertatis in segments 30-35, calciferous glands in 10-14 and a higher mean body weight (Table 3).

Etymology

Gatesona rutena was named after the Gallic tribes (the Ruten) that inhabited French lands before Roman invasion (see Bouché [26] page 5).

Material examined

Holotype

FRANCE • Adult; Occitanie, Aveyron, Rodez; 17/04/1968; leg. Marcel Bouché; Locality 1: 44.415 2.039433 Pasture; deposited in the collection MNHN (voucher: 91/619/0712).

Additional material

FRANCE • 7 specimens, 4 adults, 3 juveniles; Occitanie, Aveyron, Rodez; 10/11/2019; leg. Daniel F. Marchán; Locality 1: 44.416565 2.041011 Oak forest; deposited in the CEFE earthworm collection (voucher: 70257).

Morphological description

External morphology

Body pigmentation very faint brown, white-creamy spots can be observed through the body wall (Figure 2a). White-beige with pigmentary dots scattered along the mid-segment line in the cephalic (sometimes tail) region in fixed specimens (Figure 3b,f).

Mean length 106.5 mm (98-115 mm, n = 2 adults); body cylindrical in cross-section; mean number of segments 183 (175-192, n = 2 adults; 192 segments in the holotype). Mean weight (fixed specimens): 2.82 g (2.16-3.47 g, n = 2 adults). Prostomium epilobous, closed. Longitudinal furrows in segments 1 and 2. First dorsal pore at intersegmental furrow 4/5. Nephridial pores aligned in ~~B~~ close to ~~b~~. Spermathecal pores at intersegmental furrows 9/10 and 10/11 (plus 11/12 according to Bouché 1972 page 439) in *c*. Male pores in segment 15, surrounded by a well-developed porophore. Female pores on the posterior part of segment 14, inconspicuous. Clitellum saddle-shaped in segments 26-35. Tubercula pubertatis in segments 30-35. Chaetae small and closely paired, with interchaetal ratio *aa*: 15, *ab*: 1, *bc*: 8.5, *cd*: 0.7, *dd*: 27.5 at segment 40. Chaetophores/genital papillae in segments 12, 16, 17, 27, 24-36.

Internal anatomy

Septum 4/5 thickened, septa 5/6-8/9 strongly thickened. Hearts in segments 6-11, oesophageal. Calciferous glands in segments 10-14, dilated in segment 10. Crop in segments 15-16, gizzard in segments 17-18. Typhlosole bifid. Male sexual system holandric, testes and funnels (not enclosed in testes sacs, but with sperm present) located ventrally in segments 10 and 11. Four pairs of reniform seminal vesicles in segments 9, 10, 11 and 12, with the latter two pairs being larger. Ovaries and female funnels in segment 13, ovarian receptacles (ovisacs) in segment 14. Two pairs of globular spermathecae in segments 9 and 10 (an additional spermathecae can appear in 11 in one or both sides of the body). Nephridial bladders sigmoid, proclinate in anterior and posterior segments (Fig. 3j).

Distribution and ecology: *Gatesona rutena* is known from the region of Rodez, in the western Massif Central, France. This species was found at an altitude of 360 meters in a

prairie/oak forest transition on silty soil, with close to neutral pH (7.2), 4.79% organic matter and 0.414% nitrogen content [26].

***Gatesona lablacherensis* comb.nov stat. nov.**

Allolobophora chaetophora lablacherensis Bouché 1972

Redescription

Diagnosis

Specimens of *Gatesona lablacherensis* can be distinguished from other known species of *Gatesona* by the position of the clitellum in segments 25-32 and tubercula pubertatis in segments 27-30, as well as by possessing three pairs of seminal vesicles (in segments 10, 11 and 12) rather than four (Table 3).

Etymology

The name *lablacherensis* refer to Lablachère, the region of France the species is known to inhabit.

Holotype

FRANCE • Adult; Rhône-Alps, Ardèche, Lablachère; 20/11/1968; leg. Marcel Bouché; Locality 1: 44.433, 4.22643 Oak forest over rocky outcrops; deposited in MNHN (voucher: 90/1056/0716).

Additional material

FRANCE • 12 specimens, adults; Rhône-Alps, Ardèche, Lablachère; 6/11/2019; leg. Daniel F. Marchán; Locality 1: 44.433, 4.22643 Oak forest over rocky outcrops; deposited in the CEFE earthworm collection (voucher: 70256).

Morphological description

External morphology

Body pigmentation absent, very conspicuous white-creamy spots can be observed through the body wall (Figure 2b). White-beige with pigmentary dots scattered along the mid-segment line in the cephalic region in fixed specimens (Figure 3c,g).

Mean length 94 mm (88-100 mm, n = 2 adults); body cylindrical in cross-section; mean number of segments 189 (184-193, n = 2 adults; 193 segments in the holotype). Mean weight (fixed specimens): 1.68 g (1.64-1.72 g, n = 2 adults). Prostomium epilobous, closed. Longitudinal furrows in segments 1 and 2. First dorsal pore at intersegmental furrow 5/6. Nephridial pores aligned in B close to b. Spermathecal pores at intersegmental furrows 9/10 and 10/11 in *c*. Male pores in segment 15, surrounded by a well-developed porophore. Female pores on the posterior part of segment 14, inconspicuous. Clitellum saddle-shaped in segments 25-32. Tubercula pubertatis in segments 27-30 strongly developed as downward projections of the body wall. Chaetae small and closely paired, with interchaetal ratio *aa*: 16, *ab*: 1, *bc*: 8.5, *cd*: 1, *dd*: 31 at segment 40. Chaetophores/genital papillae in segments 13, 14, 16, 17, 19, 23-33.

Internal anatomy

Septum 4/5 thickened, septa 5/6-8/9 strongly thickened. Hearts in segments 6-11, oesophageal. Calciferous glands in segments 10-12, dilated in segment 10. Crop in segments 15-16, gizzard in segments 17-18. Typhlosole bifid. Male sexual system holandric, testes and funnels (not enclosed in testes sacs, but with sperm present) located ventrally in segments 10 and 11. Three pairs of reniform seminal vesicles in segments 10, 11 and 12, with the latter two pairs being larger. Ovaries and female funnels in segment 13, ovarian receptacles (ovisacs) in segment 14. Two pairs of

globular spermathecae in segments 9 and 10. Nephridial bladders sac-like or J shaped in the first segments, which gradually change to sigmoid, proclinate (Fig. 3k).

Distribution and ecology: *Gatesona lablacherensis* is known from the region of Lablachère, in the eastern Massif Central, France. This species has been found in an oak forest, and thus may prefer natural habitats.

This species was found at an altitude of 180 meters in a prairie/oak forest transition on silty soil over rocks, with close to neutral pH (7.6), 1.9% organic matter and 0.166% nitrogen content [26].

Gatesona serninensis comb.nov stat. nov.

Allolobophora chaetophora serninensis Bouché 1972

Redescription

Diagnosis

Specimens of *Gatesona serninensis* can be distinguished from other known species of *Gatesona* by the position of the clitellum in segments 25-33 and tubercula pubertatis in segments 28-32(33), as well as by the significantly lower number of segments (Table 3). *Gatesona serninensis* is similar to *Gatesona transmatica* in the position of tubercula pubertatis (28-32(33) vs 29-32) and they share the same position of the clitellum, but they are easily differentiated by the position of the first dorsal pore (5/6 vs (6/7) 7/8) and the number and position of seminal vesicles (4 in 9, 10, 11, 12 vs 3 in 9, 11, 12).

Etymology

The name *serninensis* refer to Saint-Sernine-sur-Rance, the town in the vicinities of whichr the species are known to inhabit.

Holotype

FRANCE • Adult; Occitanie, Aveyron, Saint-Sernin-sur-Rance; 26/11/1968; leg. Marcel Bouché; Locality 1: 43.875 2.606433 Prairie; deposited in MNHN (voucher 88/1100/0714)

Additional material

FRANCE • 12 specimens, 8 adults, 4 juveniles; Occitanie, Aveyron, Saint-Sernin-sur-Rance; 23/04/2021; leg. Daniel F. Marchán; Locality 1: 43.956, 3.218433 Prairie; deposited in the CEFE earthworm collection (voucher 70258).

Morphological description

External morphology

Body pigmentation absent, conspicuous white-creamy spots can be observed through the body wall. White-beige with pigmentary dots scattered along the mid-segment line in the cephalic (sometimes caudal) region in fixed specimens (Figure 3d,h).

Mean length 85 mm (70-99 mm n = 6 adults); body cylindrical in cross-section; mean number of segments 128 (118-147, n = 8 adults; 121 segments in the holotype). Mean weight (fixed specimens): 1.32 g (1.085-1.675 g, n = 4 adults). Prostomium epilobous, closed. Longitudinal furrows in segments 1 and 2. First dorsal pore at intersegmental furrow 5/6. Nephridial pores aligned in cd. Spermathecal pores at intersegmental furrows 9/10 and 10/11 in *c*. Male pores in segment 15, surrounded by a well-developed porophore. Female pores on the posterior part of segment 14, inconspicuous. Clitellum saddle-shaped in segments 25-33. Tubercula pubertatis in segments 28-32 (33). Chaetae

small and closely paired, with interchaetal ratio *aa*: 13.3, *ab*: 1, *bc*: 8.3, *cd*: 1, *dd*: 33 at segment 40. Chaetophores/genital papillae in segments 11, 16, 17, 19-33, those in 11, 16, 32 and 33 especially well developed and constant.

Internal anatomy

Septa 5/6-9/10 strongly thickened. Hearts in segments 6-12, oesophageal. Calciferous glands in segments 10-12, with diverticles in segment 10. Crop in segments 15-16, gizzard in segments 17-18. Typhlosole bifid. Male sexual system holandric, testes and funnels (not enclosed in testes sacs, but with sperm present) located ventrally in segments 10 and 11. Four pairs of reniform seminal vesicles in segments 9, 10, 11 and 12, with the latter two pairs being larger. Ovaries and female funnels in segment 13, ovarian receptacles (ovisacs) in segment 14. Two pairs of globular spermathecae in segments 9 and 10. Nephridial bladders J shaped in the first segments, which gradually change to sigmoid, proclinate (Fig. 3l).

Distribution and ecology: *Gatesona* ~~*lablacherensis*~~ is known from the region of Saint-Sernin-sur-Rance, in the southern Massif Central, France. This species has been found at an altitude of 350 meters in the transition between an oak-chestnut forest and a prairie on sandy soil over rocks, with acid pH (5.6), 3.78% organic matter and 0.350% nitrogen content [26].

Conclusions

Integrative systematics supported the status of the previously described subgenus *Allolobophora (Gatesona)* as a genus, including some representatives of *Helodrilus* (*Acystodrilus)*. Additionally, a clade comprising *Gatesona* and *Avelona* was recovered

by molecular phylogenetics, an early branching clade with most of its species being endemic to the Massif Central. Morphological and molecular evidence indicated most of the previously described subspecies of *Gatesona chaetophora* should actually be regarded as proper species. The presence of this diverse assemblage of endemic earthworms, with several taxa remaining to be studied by molecular methods, highlights the interest of this region of Southern France for further sampling effort across their range.

The phylogenetic distinctness of representatives of *Helodrilus (Helodrilus)* and *Helodrilus* (*Acystodrilus)* indicate that a more comprehensive sampling of species (including *He. oculatus*) is required in order to establish a solid systematic revision of the elusive genus.

Acknowledgements

We are indebted to Marcel Koken who kindly provided the specimens of *Avelona ligra*.

We are also thankful to Marcel Bouché for making the holotypes available for morphological study.

This work was supported by a grant from the Systematic Research Fund and by Xunta de Galicia. Consellería de Cultura, Educación e Ordenación Universitaria. Secretaria Xeral de Universidades under grant ED431B 2019/038.

DFM was funded by a Juan de La Cierva-Formación grant (FJCI-2017-32895) from the Spanish Ministry of Sciences, Innovation and Universities.

References

[1] Peyaud JB, Barbarand J, Carter A, Pagel M. Mid-Cretaceous uplift and erosion on the northern margin of the Ligurian Tethys deduced from thermal history reconstruction. Int J Earth Sci. 2005; 94(3), 462-474.

[2] Murienne J, Giribet G. The Iberian Peninsula: ancient history of a hotspot of mite harvestmen (Arachnida, Opiliones, Cyphophthalmi, Sironidae) diversity. Zool J Linnean Soc. 2009; 156, 785–800.

[3] Fernández R, Almodóvar A, Novo M, Gutiérrez M, Díaz Cosín DJ. Earthworms, good indicators for palaeogeographical studies? Testing the genetic structure and demographic history in the peregrine earthworm *Aporrectodea trapezoides* (Dugès, 1828) in southern Europe. Soil Biol Biochem. 2013; 58, 127-135.

[4] Novo M, Fernández R, Andrade SC, Marchán DF, Cunha L et al. Phylogenomic analyses of a Mediterranean earthworm family (Annelida: Hormogastridae). Mol Phylogenetics Evol. 2016; 94, 473-478.

[5] Domínguez J, Aira M, Breinholt JW, Stojanovic M, James SW, Pérez-Losada M. Underground evolution: new roots for the old tree of lumbricid earthworms. Mol Phylogenetics Evol. 2015; 83, 7-19.

[6] Anderson FE, Williams BW, Horn KM, Erséus C, Halanych KM, Santos SR et al. Phylogenomic analyses of Crassiclitellata support major Northern and Southern Hemisphere clades and a Pangaean origin for earthworms. BMC Evol Biol. 2017; 17(1), 123.

[7] Qiu JP, Bouché M. Révision morphologique, chorologique et taxonomique du genre Scherotheca Bouché, 1972 (Oligochaeta: Lumbricidae). Doc. Pédozool. Integrol. 1998; 4, 117-139.

[8] Qiu JP, Bouché M. Le genre Allolobophora Eisen, 1874 (Oligochaeta Lumbricidae), ses avatars et sa définition moderne. Doc. Pédozool. Integrol. 1998; 4, 86-97.

[9] Qiu JP, Bouché M. Une nouvelle évaluation du genere Helodrilus (sensu Zicsi, 1985) (Oligochaeta: Lumbricidae). Doc. Pédozool. Integrol. 1998; 4, 8-16.

[10] Pérez-Losada M., Ricoy M, Marshall JC, Domínguez J. Phylogenetic assessment of the earthworm *Aporrectodea caliginosa* species complex (Oligochaeta: Lumbricidae) based on mitochondrial and nuclear DNA sequences. Mol Phylogenetics Evol. 2009; 52 (2), 293-302.

[11] Pérez-Losada M, Breinholt JW, Aira M, Domínguez J. An updated multilocus phylogeny of the Lumbricidae (Annelida: Clitellata: Oligochaeta) earthworms. J Phylogenetics Evol Biol. 2015; 3 (1).

[12] Domínguez J, Aira M, Porto PG, Díaz Cosín DJ, Pérez-Losada M. Multigene phylogeny reveals two new isolated and relic earthworm genera (Oligochaeta: Lumbricidae). Zool J Linnean Soc. 2018; 182(2), 258-274.

[13] Pérez-Losada M, Breinholt JW, Porto PG, Aira M, Domínguez J. An earthworm riddle: systematics and phylogeography of the Spanish lumbricid *Postandrilus*. PLoS One. 2011; 6 (11).

[14] Paoletti MG, Blakemore RJ, Csuzdi C, Dorigo L, Dreon AL, Gavinelli F. et al. Barcoding *Eophila crodabepis* sp. nov. (Annelida, Oligochaeta, Lumbricidae), a large stripy earthworm from alpine foothills of northeastern Italy similar to *Eophila tellinii* (Rosa, 1888). PloS One. 2016 ; 11 (3).

[15] de Sosa I, Díaz Cosín DJ, Csuzdi C, Paoletti MG, Marchán DF. Placing *Eophila tellinii* (Oligochaeta, Lumbricidae) in a molecular phylogenetic context advances the century-old controversy around the problematic genus. Eur J Soil Biol. 2019; 94.

[16] Katoh K, Standley D.M. MAFFT multiple sequence alignment software version 7: improvements in performance and usability. Mol Biol Evol. 2013; 30 (4), 772-780.

[17] Hall TA. BioEdit: a user-friendly biological sequence alignment editor and analysis program for Windows 95/98/NT. Nucleic Acids Symposium Series. 1999; 41, 95-98.

[18] Darriba D, Taboada GL, Doallo R, Posada D. jModelTest 2: more models, new heuristics and parallel computing. Nat Methods. 2012; 9 (8), 772.

[19] Akaike H. Maximum likelihood identification of Gaussian autoregressive moving average models. Biometrika. 1973; 60 (2), 255-265.

[20] Schwarz G. Estimating the dimension of a model. Ann Stat. 1978; 6 (2), 461-464.

[21] Stamatakis A. RAxML Version 8: A tool for Phylogenetic Analysis and Post-Analysis of Large Phylogenies. Bioinformatics. 2014; 30 (9), 1312-1313.

[22] Miller MA., Pfeiffer W, Schwartz T. Creating the CIPRES Science Gateway for inference of large phylogenetic trees. Gateway Computing Environments Workshop (GCE), New Orleans, LA. 2010, pp. 1-8

[23] Ronquist F, Huelsenbeck JP. MrBayes 3: Bayesian phylogenetic inference under mixed models. Bioinformatics. 2003; 19 (12), 1572-1574.

[24] Marchán DF, Fernández R, de Sosa I, Sánchez N, Díaz Cosín DJ, Novo M. Integrative systematic revision of a Mediterranean earthworm family: Hormogastridae (Annelida, Oligochaeta). Invertebr Syst. 2018; 32 (3), 652-671.

[25] Kumar S, Stecher G, Li M, Knyaz C, Tamura K. MEGA X: molecular evolutionary genetics analysis across computing platforms. Mol Biol Evol. 2018; 35 (6), 1547-1549.

[26] Bouché MB. Lombriciens de France: écologie et systématique (Vol. 72, No. 2). Paris: Institut National de la Recherche Agronomique. 1972

[27] Szederjesi T, Pop VV, Pavlícek, Márton O, Krízsik V, Csuzdi C. Integrated taxonomy reveals multiple species in the Dendrobaena byblica (Rosa, 1893) complex (Oligochaeta: Lumbricidae). Zool J Linnean Soc. 2018; 182(3), 500-516.

[28] Cresko WA, Amores A, Wilson C, Murphy J, Currey M, Phillips P. et al. Parallel genetic basis for repeated evolution of armor loss in Alaskan threespine stickleback populations. PNAS. 2004; 101(16), 6050-6055.

[29] Szederjesi T, Angyal D, Balázs G, Dány, L. Remarks on the earthworm genus Helodrilus Hoffmeister, 1845 with new epigean and subterranean records (Oligochaeta, Lumbricidae). Opusc Zool Budapest. 2014; 45(2), 181-188.

[30] Csuzdi, C. Earthworm species, a searchable database. Opusc Zool Budapest. 2012; 43(1): 97-99

Supporting information captions

S1. Species included in the reference dataset

S2. Phylogenetic tree obtained by Bayesian phylogenetic analysis of the concatenated sequence of molecular markers *COI*–16S-tRNAs–*ND1*–28S.

S3. Uncorrected average pairwise (UAPG) distances for the marker 16S between representatives of different well-established genera of Lumbricidae.

Figure 1

Click here to access/download;Figure;Figure 1.tif

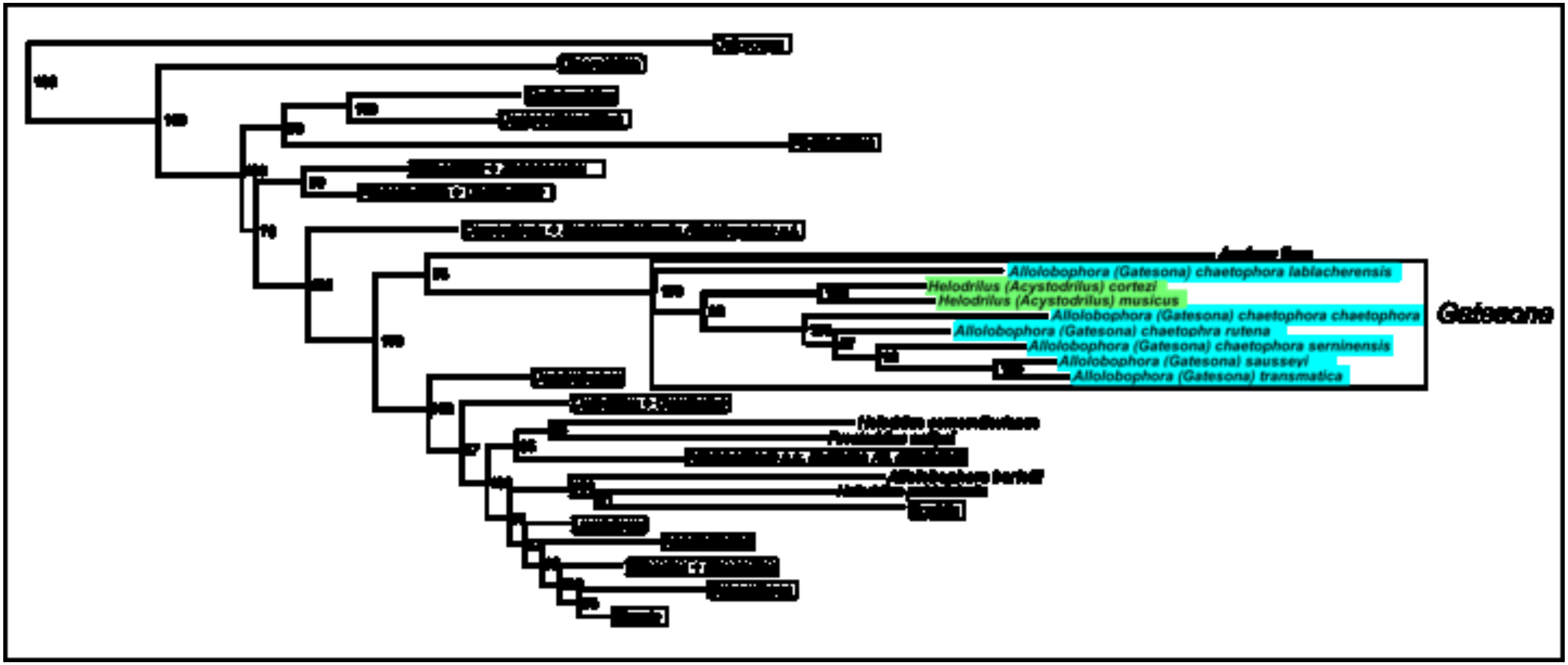

Figure 2

Click here to access/download;Figure;Figure 2.tif

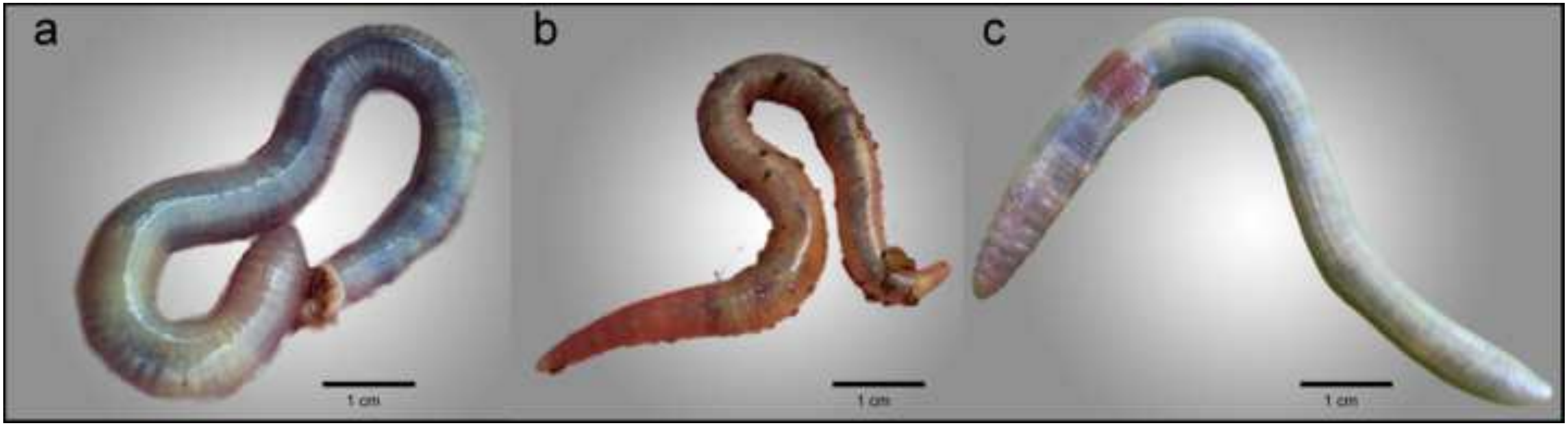

Figure 3

Click here to access/download;Figure;Figure 3.tif

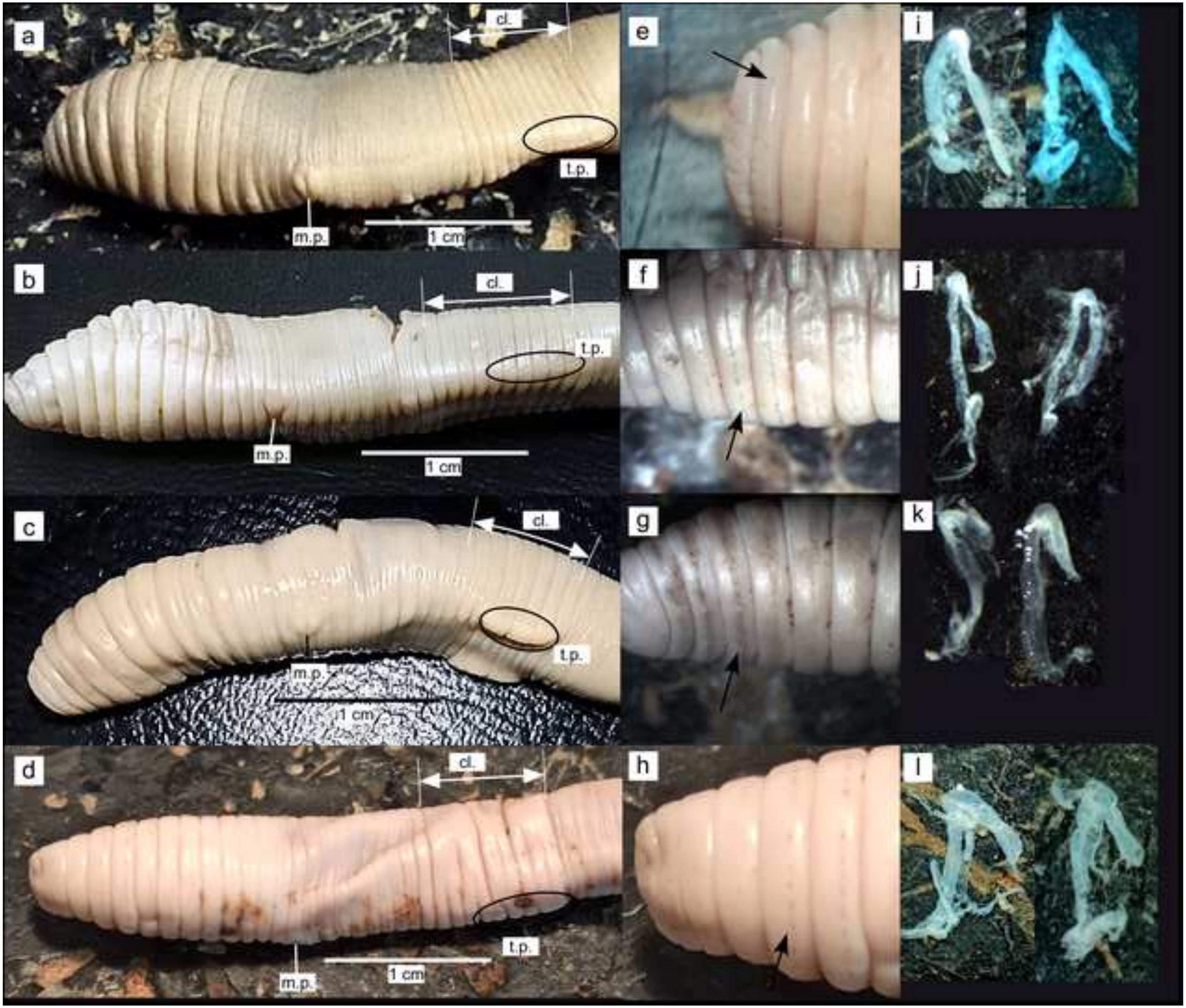

Supporting Information S1

Click here to access/download
Supporting Information
S1.docx

Supporting Information S2

Click here to access/download
Supporting Information
S2.docx

Click here to access/download
Supporting Information
S3.docx

Full title:

Systematic revision of *Gatesona* (Crassiclitellata, Lumbricidae), an endemic earthworm genus from the Massif Central (France)

Short title: Systematic revision of the earthworm genus Gatesona

Daniel F. Marchán[1,#a,]*, Sergio Jiménez[2], Thibaud Decaëns[1], Jorge Domínguez[3] Domínguez[1]

[1] CEFE, UMR 5175, CNRS–Univ Montpellier–Univ Paul–Valéry–EPHE–SupAgro Montpellier–INRA–IRD, 1919 Route de Mende, 34293, Montpellier Cedex 5, France[1] Grupo de Ecoloxía Animal (GEA), Universidade de Vigo, E-36310 Vigo, Spain

[2] Department of Biodiversity, Ecology and Evolution, Faculty of Biology, Universidad Complutense de Madrid, Madrid, Spain.

[3]Grupo de Ecoloxía Animal (GEA), Universidade de Vigo, E-36310 Vigo, Spain

[#a] Current address: CEFE, UMR 5175, CNRS–Univ Montpellier–Univ Paul–Valéry–EPHE–SupAgro Montpellier–INRA–IRD, 1919 Route de Mende, 34293, Montpellier Cedex 5, France

Formatted: English (United States)

Formatted: English (United States)

Formatted: English (United States)

Formatted: English (United States)

Formatted: English (United States)

*Corresponding author- Email address: daniel.fernandez-marchan@umontpellier.frdanifermch@gmail.com

Abstract

The Massif Central in France could potentially harbor numerous ancient endemic lineages owing to its long history of continuous geological stability. Several endemic earthworm species inhabit the area, with *Allolobophora (Gatesona) chaetophora, Helodrilus (Acystodrilus)* and *Avelona ligra* showing hints of a common evolutionary origin. However, the phylogenetic relationships and taxonomic status of the species remain to be studied through integrative molecular and morphological methods. To this end, eight species including most of the known species and subspecies of *All. (Gatesona), Helodrilus (Acystodrilus) musicus,* and *Avelona ligra* were sequenced for a set of five molecular markers. The species were grouped on the basis of the molecular findings in a phylogenetic framework. *All. (Gatesona)* was included within the same clade as *Helodrilus (Acystodrilus)* and *Avelona,* separated from *Allolobophora* sensu stricto, supporting its status as a good genus. Branch lengths and average pairwise genetic distances suggested the subspecies of *All. (Gatesona) chaetophora* examined should be considered species-level taxa. Thus, a generic diagnosis for *Gatesona* stat. nov. is provided, along with redescriptions of *Gatesona chaetophora* comb. nov., *Gatesona rutena* comb. nov.*rutena* stat. nov.,. and *Gatesona lablacherensis* comb. nov. stat. nov. and *Gatesona serninensis* comb. nov. nov.stat. nov. The study findings

Formatted: Font: Not Italic

highlight the need for further sampling of earthworm diversity in the Massif Central (and Southern France), in addition to an increased focus on the Eastern European species of *Helodrilus*.

Introduction

The Massif Central in France is an ancient massif which originated in the Variscan orogeny (Late Paleozoic) and has been an emerged continental realm through the Mesozoic and Cenozoic to the present [1]. Owing to this stability over geological time, the region is expected to harbour a large number of ancient endemic lineages (as observed for mite harvestmen in the Iberian Peninsula [2]. Earthworms, whose phylogeography and biogeography are known to reflect paleogeographic events [3], are a good example of this prediction. The Massif Central is inhabited by several endemic earthworm species from narrowly distributed genera: *Hemigastrodrilus monicae* Bouché 1970 (a basal lineage of Hormogastridae [4]), *Ethnodrilus aveli* Bouché 1972, *Ethnodrilus gatesi* Bouché 1972, *Zophoscolex graffi* Bouché 1972, *Zophoscolex micellus* Bouché 1972, *Allolobophora (Gatesona) chaetophora* Bouché 1972, *Helodrilus (Acystodrilus) cortezi* Qiu & Bouché 1998, *Helodrilus (Acystodrilus) musicus* Qiu & Bouché 1998, *Helodrilus (Acystodrilus) segalensis* Qiu & Bouché 1998 and *Avelona ligra* Bouché 1969.

Among the above-mentioned species, *All. (Ga.) chaetophora, Helodrilus (Acystodrilus)* and *Avelona ligra* may share a common evolutionary origin according to different sources of evidence. *All. (Ga.) chaetophora* and *He. (Ac.) cortezi* were grouped together in a relatively basal clade in the molecular phylogenetics analysis reported by Domínguez et al. [5]. This position is congruent with their morphological characteristics, as excepting by~~besides~~ their nephridial bladder structure (sigmoid vs absent) both taxa are very similar. *Av. ligra* is also endemic to the (northeastern) Massif Central and morphologically similar to both taxa. The only molecular phylogenetics study including *Av. ligra* [6] found that it is closely related to *All. (Ga.) chaetophora*; however, the sparse taxon sampling of Lumbricidae does not provide robust support for such a clade. In order to confirm a clade comprising these species, additional representatives of these taxa with a common molecular marker set must be examined.

In addition to the phylogenetic uncertainty, there remain some taxonomic questions regarding *All.* (*Ga.*) *chaetophora*. Bouché (1972) described several subspecies within *All. (Ga.) chaetophora* with very restricted ranges and significant morphological differences from the type species. Bouché acknowledged that such morphological heterogeneity should be delimited in further studies (which he was unable to perform). Surprisingly, this type of study was performed in Qiu and Bouché [7] for other species complexes such as *Scherotheca* -resulting in the elevation of several subspecies to species status- but not for *All. (Ga.) chaetophora*. However, two closely related species, *All. (Ga.) sausseyi* Qiu & Bouché 1998 and *All. (Ga.) transmatica* Qiu & Bouché 1998, were described and added to the subgenus *Gatesona* [8].~~.~~ The position of *Allolobophora chlorotica* (type species of *Allolobophora*) within a well-supported, independent clade from *All.* (*Ga.*) *chaetophora* [5] suggests that *Gatesona* could be erected as a proper

genus. Molecular phylogenetic analysis of additional species (and subspecies) would contribute to a robust assessment of this hypothesis.

In this study, a systematic revision of *Allolobophora (Gatesona)* is proposed based on a molecular phylogenetic analysis including several species and subspecies. In addition, the phylogenetic link between *Allolobophora (Gatesona), Helodrilus (Acystodrilus)* and *Avelona ligra* was tested by the inclusion of the later in the same phylogenetic data set.

Materials and methods

Specimens, sampling and morphological description

The specimens described in this study were collected in a sampling survey carried out in Southeastern France and Catalonia between October and November 2019, with additional sampling in Spring 2021.. The list of species and the locations where they were collected are shown in Table 1.

Earthworms were obtained by soil digging and hand-sorting, rinsed with water and fixed in 70% in order to obtain relaxed (as opposed to excessively retracted) specimens. They were subsequently transferred to 100% ethanol to enable further molecular analyses. Species classification and morphological diagnoses were conducted using the same set of external and internal morphological characters used by Qiu & Bouché [8][9]. The following main external morphological characters were considered: mean length, mean number of segments, mean weight, pigmentation, type of prostomium, setal arrangement, position of papillae, position of first dorsal pore, nephridial pore

arrangement, position and development of male pores, position and development of female pores, position of spermathecal pores, position of clitellum and position of tubercula pubertatis.

The main internal anatomical characters considered were as follows: position of oesophageal hearts, position and morphology of calciferous glands, position of crop, position of gizzard, type of typhlosole, shape of nephridial bladders, number and position of seminal vesicles, and number and position of spermathecae.

Holotypes of *Allolobophora (Gatesona) chaetophora* and its different subspecies (see table 1), temporally available at the personal collection of Marcel Bouché, were studied and compared with the newly sampled material.

Table 1. Species sampled and sequenced in this study, with their sampling location and coordinates. *Individuals from this species were newly sampled for morphological study but previously existing sequences were used for the phylogenetic analyses.

Taxon	Location	Coordinates
*Allolobophora (Gatesona) chaetophora chaetophora**	L'Hospitalet du Larzac	43.956, 3.218433
Allolobophora (Gatesona) chaetophora lablacherensis Bouché 1972	Lablachère, France	44.433, 4.22643
Allolobophora (Gatesona) chaetophora serninensis Bouché 1972	Saint-Sernin-sur-Rance	43.87487, 2.60635
Allolobophora (Gatesona) chaetophora rutena Bouché 1972	Rôdez, France	44.415, 2.03943
Allolobophora (Gatesona) sausseyi	Argelès-sur-Mer, France	42.52361, 3.04193

Formatted: Font: Not Italic

Formatted: Font: Not Italic

Formatted: Font: Not Italic

Allolobophora (Gatesona) transmatica	Agullana, Spain	42.39626, 2.84851
Helodrilus (Acystodrilus) musicus	Saint-Germain-de-Calberte, France	44.21667, 3.79999
Avelona ligra	Jargeau, FranceTo be provided	Not available To be provided
Allolobophora bartolii Bouché 1970	Jouques, France	43.641, 5.63043

DNA isolation, sequencing and molecular analyses

Total genomic DNA was extracted from ventral integument samples of approximately 5 x 5 mm by using the DNeasy Blood & Tissue Kit (Qiagen). For 8 species, regions of the nuclear 28S rRNA and mitochondrial 16S rRNA, tRNAs Ala, Leu and Ser, NADH dehydrogenase (*ND1*) and *COI* were amplified by polymerase chain reaction (PCR), with the primers and conditions described in Pérez-Losada *et al.* [10][11]. PCR products were purified and sequenced by the C.A.C.T.I Genomics service (University of Vigo).

DNA sequences obtained in this study are available in Genbank, under accession numbers MZ153163-MZ153169, MZ156573-MZ156588 and MZ235293-MZ235314XXXX-YYYYY.

Sequences reported by Domínguez *et al.* [5][12], Pérez-Losada *et al.* [10][11][13], Paoletti *et al.* [14] and de Sosa *et al.* [15], including representatives of most of the Lumbricidae genera and two members of the closest families (Hormogastridae and Criodrilidae), were downloaded from Genbank and used as a reference data set. The species included are listed in Supporting Information S1.

Sequences were aligned with MAFFT v.7 [16] with default settings and concatenated with BioEdit [17], resulting in a matrix of 3,201 bp. The best fitting evolutionary model for each partition was selected with jModelTest v. 2.1.3 [18] by applying the Akaike information criterion (AIC [19]), and Bayesian information criterion (BIC [20]). GTR +

Formatted: Font: English (United States)

Formatted: Font: English (United States)

I + G was selected as the best-fitting evolutionary model for COI, 28S and ND1, HKY+I+G was selected for 16S and HKY+G was selected for tRNAs.

Maximum Likelihood analysis was performed with RaxML-NG [21] as implemented in the CIPRES Science Gateway V. 3.3 [22], with 10 random starting trees and estimating the support for the resulting topologies with 1,000 rapid bootstrap replicates. Bayesian Inference of the phylogeny was estimated with MRBAYES v.3.1.2 [23] as implemented in the CIPRES Science Gateway V. 3.3. Parameters were set to 50 million generations and sampled every 5,000th generation (10,000 trees). Two independent runs each with four chains were performed and 20% of the trees were discarded as burn-in. The remaining trees were combined and summarized on a 50% majority-rule consensus tree. Clade support values over 70% and 90% (for Bootstrap and Posterior probability respectively) were considered high (see [15][24]).

Uncorrected average pairwise distances between the studied species for the molecular markers COI and 16S were calculated in MEGA X [25] in order to support their status as separate species and to study genetic distances within and between genera.

Results and Discussion

Molecular phylogenetic analyses

Bayesian inference (Figure 1) and maximum likelihood inference phylogenetic trees recovered consistent topologies. *Avelona ligra* and representatives of *Helodrilus (Acystodrilus)* and *Allolobophora (Gatesona)* species formed a well-supported clade, isolated from species assigned to *Allolobophora* and *Helodrilus*. *Allolobophora (Gatesona)* and *Helodrilus (Acystodrilus)* appeared intermixed in a well-supported genus-level clade, hereafter referred to as *Gatesona*. Within *Gatesona*, representatives of the different subspecies of *All.* (*Ga.) chaetophora* were separated by long branches. In addition, *Ga. sausseyi* and *Ga. transmatica* were nested within a clade including *All.* (*Ga.) chaetophora* and its different subspecies.

Figure 1. Detail of the phylogenetic tree obtained by Bayesian phylogenetic analysis of the concatenated sequence of molecular markers *COI*–16S-tRNAs–*ND1*–28S (see full tree in Supporting Information S2). *Allolobophora (Gatesona)* representatives are shaded in blue and *Helodrilus (Acystodrilus)* representatives are shaded in green. Representatives of the main Lumbricidae genera were collapsed and are shown in boxes. Posterior probability support values are shown besides the corresponding nodes.

COI and 16s uncorrected average pairwise genetic (UAPG) distances between the species of the genera *Avelona, Gatesona* (comprising *All. (Gatesona*) and *He (Acystodrilus*)) and *Helodrilus (Helodrilus)* included in this study are shown in Table 2. UAPG distances between representatives of *Avelona, Gatesona* and *Helodrilus (Helodrilus)* were high, ranging from 15.2% to 21% for COI and from 11.0% to 17.5% for 16s. UAPG distances within the genus-level clades were lower, with values corresponding to *Gatesona* (8.1%-17.3% COI, 4.1%-13.2% 16s) being generally lower

than distances within *Helodrilus (Helodrilus)* (17% COI, 11.9% 16s). UAPG distances between the different species and subspecies of *Gatesona* were high (see above) except for *All. (Ga.) sausseyi- All. (Ga.) transmatica* (8.1% COI, 4.1% 16s).

Table 2. Uncorrected average pairwise genetic (UAPG) distances between species of the genera *Avelona*, *Helodrilus (Acystodrilus)*, *Gatesona* and *Helodrilus* (*Helodrilus*) included in this study for the molecular markers COI (below the diagonal) and 16S (above the diagonal). Red shading indicates higher values and green shading indicates lower values. The COI ~~UAPG~~ distance between *All.* (*Ga.*) *rutena* and *He.* (*Ac.*) *musicus* is not displayed as it was unusually low. Asterisks indicate COI sequences were not available for the species.

	Av. ligra	*He. (Ac.) cortezi*	*He. (Ac.) musicus*	*All. (Ga.) chaetophora*	*All. (Ga.) chaetophora lab.*	*All. (Ga.) chaetophora ser.*	*All. (Ga.) chaetophora rutena*	*All. (Ga.) sausseyi*	*All. (Ga.) transmatica*	*He. cernosvitovianus*	*He. patriarchalis*
Av. ligra		14.3	16.9	16.6	13.5	12.4	13.5	14.6	15.7	13.3	13.1
He. (Ac.) cortezi	19.6		7.9	11.2	10.2	11.7	10.6	10.5	10.2	14.0	15.0
He. (Ac.) musicus	19.6	10.6		12.5	12.5	13.1	12.0	9.7	11.6	14.8	16.3
All. (Ga.) chaetophora	15.2	16.0	16.0		13.2	11.3	9.1	11.3	13.2	13.6	14.6
All. (Ga.) chaetophora lablacherensis	*	*	*	*		10.1	9.0	9.7	10.4	11.4	14.2
All. (Ga.) chaetophora serninensis	19.0	16.7	16.2	14.5	*		8.2	6.7	9.0	14.0	17.5
All. (Ga.) chaetophora rutena	17.8	9.1		13.6	*	14.6		7.5	7.9	11.0	13.5
All. (Ga.) sausseyi	18.3	17.3	16.5	14.4	*	14.5	14.1		4.1	14.8	17.1
All. (Ga.) transmatica	18.5	16.7	16.3	13.2	*	12.9	14.1	8.1		14.0	16.3
He. cernosvitovianus	20.5	19.0	18.8	17.5	*	19.5	17.3	19.8	19.1		11.9
He. patriarchalis	19.1	18.6	18.8	17.7	*	20.1	17.2	18.2	18.2	17.0	

Formatted: Font: Bold

Morphological analysis

The representatives of *Helodrilus (Acystodrilus) musicus* examined were wholly compatible with the available description [8] and did not differ in any morphological character.

External and internal morphological characters of the species and subspecies of *Allolobophora* (*Gatesona)* and related *Helodrilus (Acystodrilus)* species are shown in Table 3. Specimens were consistent with the holotypes and available descriptions [8][9][26] except for the aspects detailed below.

Specimens of *All*. (*Ga.)* *rutena* differed from the previous description in the number and position of spermathecal pores (2 pairs in 9/10, 10/11 vs 3 pairs in 9/10,10/11 and 11/12). However, the holotype of *All*. (*Ga.)* *rutena* showed 3 pairs of spermathecae on the right side but 2 pairs of spermathecae on the left side. Thus, this character appears to show variability even within the same individual.

Formatted: Font: Not Italic

Table 3. Main morphological characters of the species and subspecies of *Allolobophora* (*Gatesona)* and related *Helodrilus (Acystodrilus)* species under study. Species indicated with *: information from Bouché [26] and Qiu and Bouché [9]. In bold, morphological characters for which differences were observed relative to the original description [26].

Characters	All. (Ga.) chaetophora*	All. (Ga.) chaetophora lablacherensis	All. (Ga.) chaetophora serninensis*	All. (Ga.) chaetophora rutena	All. (Ga.) sausseyi	All. (Ga.) transmatica	Helodrilus (Ac.) cortezi*	Helodrilus (Ac.) musicus
Length	68-85170-190 mm	88-100 mm	70-99 mm	98-115 mm	86 mm	66 mm	152-192 mm	127 mm
N. segments	190122-212	184-193	118-147	175-192	183	184	185-191	197
Weight	1.6-2.04-3.18 g	1.64-1.72g	1.085-1.675 g	2.16-3.47 g	1.25 g	1.03 g	1.10-1.36 g	1.42 g
Pigmentation	Pigmentary dots in cephalic segments	Pigmentary dots in cephalic (and tail) segments	Pigmentary dots -	Pigmentary dots in cephalic (and tail) segments	Pigmentary dots in cephalic (and tail) segments	Pigmentary dots in cephalic (and tail) segments	Pigmentary dots	**Pigmentary dots**
Setal arrangement	10:1.25:9.5:1:12.5	16:1:8.5:1:31	13.3:1:8.3:1:33–	15:1:8.5:0.7:27.5	48:-1:8:1:12	20:1:10:1:18	11.6:1.6:8.33:1:30	7:1:4:1:22
Prostomium	Epilobous	Epilobous	-	Epilobous	Epilobous	Epilobous		
Setae	Closely paired	Closely paired	-	Closely paired	Closely paired	Closely paired		
First dorsal pore	(4/5)5/6	5/6	5/6-	4/5	6/7	(6/7)7/8	4/5	4/5
Papillae	(11, 14) 16 (17, 20, 21, 23, 26) 27-36.	13,14,16,17,19,23-33	–	12,16,17,24-36	18,23-33	11,17-18,23-33		
Nephridial pores	En solfège	En solfège	–	En solfège	En solfège	En solfège		
Male pores	1/2 15, *b*	1/2 15, *b*	–	1/2 15, *b*	1/2 15, *b*	1/2 15, *b*		
Female pores	3/4 14, inconspicuous	3/4 14, inconspicuous	-	3/4 14, inconspicuous	1/2 14, b	1/2 14, b		

Formatted
Formatted
Formatted Table
Formatted
Formatted
Formatted
Formatted
Formatted
Formatted
Formatted
Formatted
Formatted
Formatted
Formatted
Formatted
Formatted
Formatted
Formatted
Formatted
Formatted
Formatted
Formatted
Formatted
Formatted
Formatted
Formatted
Formatted
Formatted Table
Formatted
Formatted
Formatted
Formatted
Formatted Table
Formatted
Formatted

Spermathecal pores	9/10, 10/11 simple in C	9/10, 10/11 simple in C	9/10, 10/11 simple in C	**9/10, 10/11 simple in C**	9/10, 10/11 simple in C	9/10, 10/11 simple in C	9/10, 10/11 simple in C	9/10, 10/11 simple in C
Clitellum	(26)27-36	25-32	25-3332	26-35	24-33	25-33	1/2 22-33	23-32
Tubercula pubertatis	(29)3026**)31-36	27-30, strongly developed	28-32(33)	30-35	28-32	29-32	28-30	27-1/2 31
Oesophageal hearts	54-11	6-11	6-12—	6-11	6-11	6-11	6-11	6-11
Calciferous glands	10-11(12, dilated in 10)	10-12, dilated in 10	10-12, diverticles in 10 –	10-14, dilated in 10	10-14, diverticles in 10	10-12	10-14	½ 10-14, diverticles in 10
Typhlosole	Bifid	Bifid	Bifid-	Bifid	Bifid	Bifid	Bifid	Bifid
Nephridial bladders	Sigmoid, proclinate	Sigmoid, proclinate	Sigmoid, proclinate–	Sigmoid, proclinate	Sigmoid, proclinate	Sigmoid, proclinate	Absent	Absent
Seminal vesicles	9<10<11,<12	10,11,12	9,10<11,12	9,10<11,12	9,10<11,12	9,11,12	9,10<11,12	9,10<11,12
Spermathecae	Simple, globular in 9,10	Simple, globular in 9,10	Simple, globular in 9,10	**Simple, globular in 9,10**	Simple, globular in 9,10	Simple, globular in 9,10	Simple, globular in 9,10	Simple, globular in 9,10
~~Crop~~	~~15-16~~	~~15-16~~	~~15-16~~	~~15-16~~	~~15-16~~	~~15-16~~		
~~Gizzard~~	~~17-18~~	~~17-18~~	~~17-18~~	~~17-18~~	~~17-18~~	~~17-18~~		

~~No mature individuals from the putative population of *All.* (*Ga.*) *serninensis* were collected, thus precluding comparison of their morphology with the description provided by Bouché [26].~~

~~Specimens of *All.* (*Ga.*)~~

Formatted
Formatted
Formatted Table
Formatted
Formatted
Formatted
Formatted
Formatted
Formatted
Formatted
Formatted
Formatted
Formatted
Formatted
Formatted
Formatted
Formatted
Formatted
Formatted
Formatted
Formatted
Formatted Table
Formatted
Formatted
Formatted
Formatted
Formatted
Formatted
Formatted
Formatted
Formatted
Formatted
Formatted

Specimens of *Helodrilus (Ac.) musicus* did not show red pigmentation as stated by [9], but displayed a faint reddish hue instead.

Nephridial pores were defined by [8][9][26] as irregularly distributed; however, newly captured material showed aligned nephridial pores.

~~*lablacherensis* differed from the previous description in the absence of seminal vesicles in segment 9; however, these vesicles were expected to be very small (according to the previous information) and can sometimes be difficult to identify due to regression or incipient development.~~

~~Specimens of *All. (Ga.) rutena* differed from the previous description in the number and position of spermathecae (2 pairs in 9/10, 10/11 vs 3 pairs in 9/10, 10/11 and 11/12).~~

Formatted: Top: 0.98", Bottom: 0.98", Header distance from edge: 0.49", Footer distance from edge:

Systematic implications

The phylogenetic position of representatives of *Gatesona* as a well-supported clade unrelated to the generotype of *Allolobophora* (*All. chlorotica*) suggests it should be considered its own genus. The inclusion of *Helodrilus (Acystodrilus) cortezi* and *Helodrilus (Acystodrilus) musicus* within this clade suggest that they also belong to *Gatesona*~~the same genus~~. The latter two species and the other representatives of *Gatesona* are very similar in most external and internal morphological characters, except for the absence of nephridial vesicles. This character appears in several unrelated lumbricid lineages (Erséus, Csudzi pers. comm.) and is thus homoplasic: the same is observed for the posterior displacement of the male pore, which has stopped being regarded as a key taxonomic character in lumbricid systematics [12][27]. Regression of a complex morphological structure is easier in evolutionary terms than the evolution of such structures [28], so it is likely that loss of nephridial bladders happened repeatedly associated with high moisture environments [29].

Another~~Other~~ species placed within *Helodrilus (Acystodrilus*) by Qiu and Bouché [9~~8~~] and not included in this work, *Helodrilus (Acystodrilus*) ~~(~~*segalensis*, ~~*putricola, tebra* and *rifensis*)~~ could be expected to belong to the same genus-level clade as *Helodrilus (Acystodrilus*) *cortezi* and *Ac. musicus*, due to their morphological similarity and geographic proximity (100-120 km~~Western European distribution (Southern France and Morocco~~); however, molecular phylogenetics analyses of this~~the~~ species ~~involved~~ is necessary to confirm this hypothesis. Furthermore, *Helodrilus putricola* and *tebra* have been found to belong to an unrelated clade (personal communication) and *Helodrilus rifensis* is considered a synonym of *Eiseniella neapolitana* (Orley 1885) [30].

The name *Helodrilus* should be restricted to the clade containing *He. oculatus*, its generotype. However, *He. oculatus* was not included in this study, whereas only two Eastern European *Helodrilus (Helodrilus)* representatives (*Helodrilus patriarchalis* and *Helodrilus cernosvitovianus*) among its 18 species (http://taxo.drilobase.org/) were included. Thus, a well-supported systematic revision of *Helodrilus* should include a more comprehensive sampling of both groups in a molecular phylogenetic framework.

As *Avelona ligra* was recovered~~Recovered~~ as a sister clade to *Gatesona*, it could be argued that it~~*Avelona*~~ should also be included within the same genus. However, the branch lengths separating both taxa in the phylogenetic tree were indicative of separate genera. The 16s UAPG distances (which are less saturated at above-species level than COI distances) between *Avelona ligra* and representatives of *Gatesona* were significantly higher than those found within *Gatesona*, and were within the range observed between well-established earthworm genera (8.6%-20.01%, average 13.4%; Supporting Information S2). In addition, *Avelona ligra* (only known species within *Avelona*) possesses only two pairs of seminal vesicles in 11, 12 (vs 3 or 4 pairs in (9,10) 11, 12) and displays striking bioluminescence.

The phylogenetic relationships, branch lengths and UAPG distances between the different species and subspecies of *Gatesona* suggest that they should all be considered different species. The existing morphological differences between these taxa, of similar or higher magnitude than those used by Qiu and Bouché [8~~9~~] to distinguish All. (*Ga.) sausseyi* and All. (*Ga.) transmatica*, support their status as proper species. ~~This includes *All. (Ga.) chaetophora serninensis*: even though no mature specimens were not available for examination, the morphological differences with other *chaetophora* subspecies indicated by Bouché [26] together with the genetic differentiation observed are consistent with its recognition as a species.~~

All. (Ga.) argentatensis could not be sampled for this study, but following the same criteria it is very likely that it constitutes an independent species from *All.* (*Ga.) chaetophora*.

Proposed systematic changes

Phylum Annelida Lamarck, 1802

Class Oligochaeta Grube, 1850/Clitellata Michaelsen, 1919

Order Crassiclitellata Jamieson, 1988~~Order Megadrili Benham, 1890/Haplotaxida Michaelsen, 1900~~

Family Lumbricidae Rafinesque-Schmaltz, 1815

Genus *Gatesona* Qiu and Bouché 1998 stat. nov.

Allolobophora (Gatesona) Qiu and Bouché 1998

Type species

Allolobophora chaetophora Bouché 1972

Included species

Gatesona chaetophora comb. nov. (Bouché 1972)

Gatesona chaetophora argentatensis comb.nov. (Bouché 1972)

Gatesona rutena comb.nov. stat. nov. (Bouché, 1972)

Gatesona lablacherensis comb.nov. (Bouché, 1972)

Gatesona serninensis comb.nov. stat. nov. (Bouché 1972)

Gatesona sausseyi comb. nov. (Qiu and Bouché 1998)

Gatesona transmatica comb. nov. (Qiu and Bouché 1998).

Gatesona cortezi comb. nov. (Qiu and Bouché 1998).

Gatesona musica comb. nov. (Qiu and Bouché 1998).

Diagnosis

Mid-sized (66 to 190 mm) Lumbricidae, with fewer than 230 segments. Mid-segment pigmentary dots in cephalic (sometimes tail) segments. Numerous chaetophores in the shape of genital papillae between segments 9 and the end of the clitellum. Prostomium epylobous. Closely paired setae. First dorsal pore in 4/5 (3 species), 5/6 (3 species), 6/7 (1 species), ~~-~~7/8 (1 species).~~.~~ Nephridial pores aligned.~~“en solfège” (irregularly distributed).~~ Male pores in ½ 15 with developed porophore. Spermathecal pores simple, in 9/10, 10/11 (exceptionally 9/10, 10/11, 11/12). Clitellum starts between segments 23 to 27. Calciferous gland variable, in 10-12(14) usually dilated or with diverticles in 10. Typhlosole bifid. Three to four pairs of seminal vesicles in (9, 10) 11, 12. Nephridial vesicles sigmoid, proclinate, sometimes absent (*Ga. cortezi*, *Ga. musica*).

Remarks

Gatesona chaetophora argentatensis (Bouché 1972) remains a subspecies of *Gatesona chaetophora* owing to failure to include representatives in the integrative systematic revision. However, it is to be expected that *Ga. chaetophora argentatensis* could represent an independent species.

~~No sexually mature specimens of *Gatesona serninensis comb.nov.* stat. nov.~~The name *Gatesona* was chosen over *Acystodrilus* for the genus encompassing species of both former subgenera for two main reasons: first, currently there are more species of the former *Allolobophora* (*Gatesona*) than of *Helodrilus* (*Acystodrilus*); second, the name *Acystodrilus* (meaning "without vesicle") does not represent the more common character state within the redefined genus *Gatesona*.

Formatted: Font: Not Italic

Gatesona chaetophora comb.nov stat. nov.

Allolobophora chaetophora chaetophora Bouché 1972

Redescription

Diagnosis

Specimens of *Gatesona chaetophora* can be distinguished from other known species of *Gatesona* by the position of the clitellum in segments 26-36 and tubercula pubertatis in segments (29)30-36 (Table 3).

Etymology

The name *chaetophora* refers to the multiple chaetophores (glandular structures surrounding genital chaetae) this species possess.

Material examined

Holotype

FRANCE • Adult; Occitanie, Aveyron, L'Hospitalet du Larzac; 11/03/1968; leg. Marcel Bouché; Locality 1: 43.956 3.218433 Prairie; deposited in the collection of the Muséum National d'Histoire Naturelle de Paris (MNHN) (voucher: 87/550/0711).

Additional material

FRANCE • 2 specimens, adults; Occitanie, Aveyron, L'Hospitalet du Larzac; 11/02/2021; leg. (Bouché 1972) were able to be examined in this study. Thus, this species was not redescribed as was the case for the other elevated species: until further specimens are collected and examined, the morphological characters shown in Table 3 (from Bouché, 1972) should be used for identification purposes.

Daniel F. Marchán; Locality 1: 43.956, 3.218433 Prairie; deposited in the CEFE earthworm collection (voucher: DFM-0676 – DFM-0677).

Morphological description

External morphology

Body pigmentation faint brown. Beige to faint brown with pigmentary dots scattered along the mid-segment line in the cephalic region in fixed specimens (Figure 3a,e).

Mean length 76.5 mm (68-85 mm, n = 2 adults); body cylindrical in cross-section; mean number of segments 190 (n = 2 adults; 190 segments in the holotype). Mean weight (fixed specimens): 2.60 g (2.035-3.175 g, n = 2 adults). Prostomium epilobous, closed.

Longitudinal furrows in segments 1 and 2. First dorsal pore at intersegmental furrow 45/6. Nephridial pores aligned in B close to b. Spermathecal pores at intersegmental furrows 9/10 and 10/11 in *c*. Male pores in segment 15, surrounded by a well-developed porophore. Female pores on the posterior part of segment 14, inconspicuous. Clitellum saddle-shaped in segments 26-36. Tubercula pubertatis in segments (29)30-36. Chaetae small and closely paired, with interchaetal ratio *aa*: 10, *ab*: 1.25, *bc*: 9.5, *cd*: 1, *dd*: 12.5 at segment 40. Chaetophores/genital papillae in segments 11, (14, 16, 17, 18) 20, 25-28, (29), 32-36 with those in 11, 20, 25-28 and 35,36 especially well developed and constant.

Figure 2. External morphology of living specimens of a) *Gatesona rutena* b) *Gatesona lablacherensis* c) *Gatesona serninensis.*

Figure 3. External and internal morphology of fixed specimens of a) *Gatesona chaetophora* b) *Gatesona rutena* c) *Gatesona lablacherensis* d) *Gatesona serninensis.* The insets show a detailed view of the pigmentary dots characteristic of *Gatesona* species (e, f, g, h), and their nephridial bladders (i, j, k, l). Abbreviations: cl. = clitellum, m. p. = male pore, t. p.= tubercula pubertatis.

Internal anatomy

Septa 5/6-9/10 strongly thickened. Hearts in segments 5-11, oesophageal. Calciferous glands in segments 10-12, dilated in segment 10. Crop in segments 15-16, gizzard in segments 17-18. Typhlosole bifid. Male sexual system holandric, testes and funnels (not enclosed in testes sacs, but with sperm present) located ventrally in segments 10 and 11. Four pairs of reniform seminal vesicles in segments 9, 10, 11 and 12, with the latter two pairs being larger. Ovaries and female funnels in segment 13, ovarian receptacles

(ovisacs) in segment 14. Two pairs of globular spermathecae in segments 9 and 10. Nephridial bladders sigmoid, proclinate in anterior and posterior segments (Fig. 3i).

Distribution and ecology: *Gatesona chaetophora* is known from the region of Larzac in the southern Massif Central, France. This species was found at an altitude of 710 meters in a prairie on silty soil, with close to neutral pH (7.4), 2.83% organic matter and 0.308% nitrogen content [26].

Gatesona rutena comb.nov stat. nov.

Allolobophora chaetophora rutena Bouché 1972

Redescription

Diagnosis

Specimens of *Gatesona rutena* can be distinguished from other known species of *Gatesona* by the position of the first dorsal pore in 4/5, the position of the clitellum in segments 26-35 and tubercula pubertatis in segments 30-35, calciferous glands in 10-14 and a higher mean body weight (Table 3).

Etymology

Gatesona rutena was named after the Gallic tribes (the Ruten) that inhabited French lands before Roman invasion (see Bouché [26] page 5).~~The name *rutena* refers to the toponym of Rodez, the region of France the species are known to inhabit.~~

Material examined

Holotype

FRANCE • Adult; Occitanie, Aveyron, Rodez; 17/04/1968~~10/11/2019~~; leg. ~~Daniel F.~~ Marcel Bouché~~Marchán~~; Locality 1: 44.415~~416565~~ 2.039433 Pasture~~041011~~

~~Oak forest~~; deposited in the collection MNHN~~UCMLT~~ (voucher: 91/619/0712~~70257-1~~).

Additional material~~Paratypes~~

FRANCE • 7 specimens, 4 adults, 3 juveniles; Occitanie, Aveyron, Rodez; 10/11/2019; leg. ~~FRANCE • 6 specimens; same data as holotype. Deposited as follows: 6 specimens in UCMLT (vouchers: 70257-2,7)~~

Daniel F. Marchán; Locality 1: 44.416565 2.041011 Oak forest; deposited in the CEFE earthworm collection (voucher: 70257).

Morphological description

External morphology

Body pigmentation very faint brown, white-creamy spots can be observed through the body wall (Figure 2a).~~.~~ White-beige with pigmentary dots scattered along the mid-segment line in the cephalic (sometimes tail) region in fixed specimens (Figure 3b,f~~2a,c~~).

Mean length 106.5 mm (98-115 mm, n = 2 adults); body cylindrical in cross-section; mean number of segments 183 (175-192, n = 2 adults; 192 segments in the holotype). Mean weight (fixed specimens): 2.82 g (2.16-3.47 g, n = 2 adults). Prostomium epilobous, closed. Longitudinal furrows in segments 1 and 2. First dorsal pore at intersegmental furrow 4/5. Nephridial pores aligned in B close to b.~~“en solfège” (irregularly distributed).~~ Spermathecal pores at intersegmental furrows 9/10 and 10/11 (plus 11/12 according to Bouché 1972 page 439) in *c*. Male pores in segment 15, surrounded by a well-developed porophore. Female pores on the posterior part of segment 14, inconspicuous. Clitellum saddle-shaped in segments 26-35. Tubercula

pubertatis in segments 30-35. Chaetae small and closely paired, with interchaetal ratio *aa*: 15, *ab*: 1, *bc*: 8.5, *cd*: 0.7, *dd*: 27.5 at segment 40. Chaetophores/genital papillae in segments 12, 16, 17, 27, 24-36.

Internal anatomy

Septum 4/5 thickened, septa 5/6-8/9 strongly thickened. Hearts in segments 6-11, oesophageal. Calciferous glands in segments 10-14, dilated in segment 10. Crop in segments 15-16, gizzard in segments 17-18. Typhlosole bifid. Male sexual system holandric, testes and funnels (not enclosed in testes sacs, but with sperm present) located ventrally in segments 10 and 11. Four pairs of reniform seminal vesicles in segments 9, 10, 11 and 12, with the latter two pairs being larger. Ovaries and female funnels in segment 13, ovarian receptacles (ovisacs) in segment 14. Two pairs of globular spermathecae in segments 9 and 10 (an additional spermathecae can appear in~~plus~~ 11 in one or both sides of the body).~~according to Bouché 1972).~~ Nephridial bladders sigmoid, proclinate in anterior and posterior segments (Fig. 3j~~2c~~).

Distribution and ecology: *Gatesona rutena* is known from the region of Rodez, in the western Massif Central, France. This species was found at~~in~~ an altitude of 360 meters in a prairie/oak forest transition on silty soil, with close to neutral pH (7.2), 4.79% organic matter~~,~~ and 0.414% nitrogen content [26].~~it thus may prefer natural habitats.~~

~~Figure 2~~

Gatesona lablacherensis comb.nov stat. nov.

Allolobophora chaetophora lablacherensis Bouché 1972

Redescription

Diagnosis

Specimens of *Gatesona lablacherensis* can be distinguished from other known species of *Gatesona* by the position of the ~~first dorsal pore in 4/5,~~ clitellum in segments 25-32 and tubercula pubertatis in segments 27-30, as well as by possessing three pairs of seminal vesicles (in segments 10, 11 and 12) rather than four (Table 3).

Etymology

The name *lablacherensis* refer to Lablachère, the region of France the species is~~are~~ known to inhabit.

Holotype

FRANCE • Adult; Rhône-Alps, Ardèche, Lablachère; 20~~6~~/11/1968~~2019~~; leg. ~~Daniel F.~~ Marcel Bouché~~Marchán~~; Locality 1: 44.433, 4.22643 Oak forest over rocky outcrops; deposited in MNHN~~UCMLT~~ (voucher: 90/1056/0716~~70256-1~~).

Additional material~~Paratypes~~

FRANCE • 12 specimens, adults; Rhône-Alps, Ardèche, Lablachère; 6/11/2019; leg. Daniel F. Marchán; Locality 1: 44.433, 4.22643 Oak forest over rocky outcrops; deposited in the CEFE earthworm collection (voucher: 70256).

~~FRANCE • 11 specimens; same data as holotype. Deposited as follows: 11 specimens in UCMLT (vouchers: 70256-2,12)~~

Morphological description

External morphology

Body pigmentation absent, very conspicuous white-creamy spots can be observed through the body wall (Figure 2b).~~.~~ White-beige with pigmentary dots scattered along the mid-segment line in the cephalic region in fixed specimens (Figure 3c,g~~2b,d~~).

Formatted: Font color: Red

Mean length 94 mm (88-100 mm, n = 2 adults); body cylindrical in cross-section; mean number of segments 189 (184-193, n = 2 adults; 193 segments in the holotype). Mean weight (fixed specimens): 1.68 g (1.64-1.72 g, n = 2 adults). Prostomium epilobous, closed. Longitudinal furrows in segments 1 and 2. First dorsal pore at intersegmental furrow 5/6. Nephridial pores aligned in B close to b.~~"en solfège" (irregularly distributed).~~ Spermathecal pores at intersegmental furrows 9/10 and 10/11 in *c*. Male pores in segment 15, surrounded by a well-developed porophore. Female pores on the posterior part of segment 14, inconspicuous. Clitellum saddle-shaped in segments 25-32. Tubercula pubertatis in segments 27-30 strongly developed as downward projections of the body wall. Chaetae small and closely paired, with interchaetal ratio *aa*: 16, *ab*: 1, *bc*: 8.5, *cd*: 1, *dd*: 31 at segment 40. Chaetophores/genital papillae in segments 13, 14, 16, 17, 19, 23-33.

Internal anatomy

Septum 4/5 thickened, septa 5/6-8/9 strongly thickened. Hearts in segments 6-11, oesophageal. Calciferous glands in segments 10-12, dilated in segment 10. Crop in segments 15-16, gizzard in segments 17-18. Typhlosole bifid. Male sexual system holandric, testes and funnels (not enclosed in testes sacs, but with sperm present) located ventrally in segments 10 and 11. Three pairs of reniform seminal vesicles in segments 10, 11 and 12, with the latter two pairs being larger. Ovaries and female funnels in segment 13, ovarian receptacles (ovisacs) in segment 14. Two pairs of

globular spermathecae in segments 9 and 10. Nephridial bladders sac-like or J shaped in the first segments, which gradually change to sigmoid, proclinate (Fig. 3k2d).

Distribution and ecology: *Gatesona lablacherensis* is known from the region of Lablachère, in the eastern Massif Central, France. This species has been found in an oak forest, and thus may prefer natural habitats.

This species was found at an altitude of 180 meters in a prairie/oak forest transition on silty soil over rocks, with close to neutral pH (7.6), 1.9% organic matter and 0.166% nitrogen content [26].

Gatesona serninensis comb.nov stat. nov.

Allolobophora chaetophora serninensis Bouché 1972

Redescription

Diagnosis

Specimens of *Gatesona serninensis* can be distinguished from other known species of *Gatesona* by the position of the clitellum in segments 25-33 and tubercula pubertatis in segments 28-32(33), as well as by the significantly lower number of segments (Table 3). *Gatesona serninensis* is similar to *Gatesona transmatica* in the position of tubercula pubertatis (28-32(33) vs 29-32) and they share the same position of the clitellum, but they are easily differentiated by the position of the first dorsal pore (5/6 vs (6/7) 7/8) and the number and position of seminal vesicles (4 in 9, 10, 11, 12 vs 3 in 9, 11, 12).

Etymology

The name *serninensis* refer to Saint-Sernine-sur-Rance, the town in the vicinities of whichr the species are known to inhabit.

Holotype

FRANCE • Adult; Occitanie, Aveyron, Saint-Sernin-sur-Rance; 26/11/1968; leg. Marcel Bouché; Locality 1: 43.875 2.606433 Prairie; deposited in MNHN (voucher 88/1100/0714)

Additional material

FRANCE • 12 specimens, 8 adults, 4 juveniles; Occitanie, Aveyron, Saint-Sernin-sur-Rance; 23/04/2021; leg. Daniel F. Marchán; Locality 1: 43.956, 3.218433 Prairie; deposited in the CEFE earthworm collection (voucher 70258).

Morphological description

External morphology

Body pigmentation absent, conspicuous white-creamy spots can be observed through the body wall. White-beige with pigmentary dots scattered along the mid-segment line in the cephalic (sometimes caudal) region in fixed specimens (Figure 3d,h).

Mean length 85 mm (70-99 mm n = 6 adults); body cylindrical in cross-section; mean number of segments 128 (118-147, n = 8 adults; 121 segments in the holotype). Mean weight (fixed specimens): 1.32 g (1.085-1.675 g, n = 4 adults). Prostomium epilobous, closed. Longitudinal furrows in segments 1 and 2. First dorsal pore at intersegmental furrow 5/6. Nephridial pores aligned in cd. Spermathecal pores at intersegmental furrows 9/10 and 10/11 in *c*. Male pores in segment 15, surrounded by a well-developed porophore. Female pores on the posterior part of segment 14, inconspicuous. Clitellum saddle-shaped in segments 25-33. Tubercula pubertatis in segments 28-32 (33). Chaetae

small and closely paired, with interchaetal ratio *aa*: 13.3, *ab*: 1, *bc*: 8.3, *cd*: 1, *dd*: 33 at segment 40. Chaetophores/genital papillae in segments 11, 16, 17, 19-33, those in 11, 16, 32 and 33 especially well developed and constant.

Internal anatomy

Septa 5/6-9/10 strongly thickened. Hearts in segments 6-12, oesophageal. Calciferous glands in segments 10-12, with diverticles in segment 10. Crop in segments 15-16, gizzard in segments 17-18. Typhlosole bifid. Male sexual system holandric, testes and funnels (not enclosed in testes sacs, but with sperm present) located ventrally in segments 10 and 11. Four pairs of reniform seminal vesicles in segments 9, 10, 11 and 12, with the latter two pairs being larger. Ovaries and female funnels in segment 13, ovarian receptacles (ovisacs) in segment 14. Two pairs of globular spermathecae in segments 9 and 10. Nephridial bladders J shaped in the first segments, which gradually change to sigmoid, proclinate (Fig. 3l).

Distribution and ecology: *Gatesona lablacherensis* is known from the region of Saint-Sernin-sur-Rance, in the southern Massif Central, France. This species has been found at an altitude of 350 meters in the transition between an oak-chestnut forest and a prairie on sandy soil over rocks, with acid pH (5.6), 3.78% organic matter and 0.350% nitrogen content [26].

Conclusions

Integrative systematics supported the status of the previously described subgenus *Allolobophora (Gatesona)* as a genus, including some representatives of *Helodrilus* (*Acystodrilus*). Additionally, a clade comprising *Gatesona* and *Avelona* was recovered

by molecular phylogenetics, an early branching clade with most of its species being endemic to the Massif Central. Morphological and molecular evidence indicated most of the previously described subspecies of *Gatesona chaetophora* should actually be regarded as proper species. The presence of this diverse assemblage of endemic earthworms, with several taxa remaining to be studied by molecular methods, highlights the interest of this region of Southern France for further sampling effort across their range.

The phylogenetic distinctness of representatives of *Helodrilus (Helodrilus)* and *Helodrilus* (*Acystodrilus)* indicate that a more comprehensive sampling of species (including *He. oculatus*) is required in order to establish a solid systematic revision of the elusive genus.

Acknowledgements

We are indebted to Marcel Koken who kindly provided the specimens of *Avelona ligra*.

We are also thankful to Marcel Bouché for making the holotypes available for morphological study.

This work was supported by a grant from the Systematic Research Fund and by Xunta de Galicia. Consellería de Cultura, Educación e Ordenación Universitaria. Secretaria Xeral de Universidades under grant ED431B 2019/038.

DFM was funded by a Juan de La Cierva-Formación grant (FJCI-2017-32895) from the Spanish Ministry of Sciences, Innovation and Universities.

References

[1] Peyaud JB, Barbarand J, Carter A, Pagel M. Mid-Cretaceous uplift and erosion on the northern margin of the Ligurian Tethys deduced from thermal history reconstruction. Int J Earth Sci. 2005; 94(3), 462-474.

[2] Murienne J, Giribet G. The Iberian Peninsula: ancient history of a hotspot of mite harvestmen (Arachnida, Opiliones, Cyphophthalmi, Sironidae) diversity. Zool J Linnean Soc. 2009; 156, 785–800.

[3] Fernández R, Almodóvar A, Novo M, Gutiérrez M, Díaz Cosín DJ. Earthworms, good indicators for palaeogeographical studies? Testing the genetic structure and demographic history in the peregrine earthworm *Aporrectodea trapezoides* (Dugès, 1828) in southern Europe. Soil Biol Biochem. 2013; 58, 127-135.

[4] Novo M, Fernández R, Andrade SC, Marchán DF, Cunha L et al. Phylogenomic analyses of a Mediterranean earthworm family (Annelida: Hormogastridae). Mol Phylogenetics Evol. 2016; 94, 473-478.

[5] Domínguez J, Aira M, Breinholt JW, Stojanovic M, James SW, Pérez-Losada M. Underground evolution: new roots for the old tree of lumbricid earthworms. Mol Phylogenetics Evol. 2015; 83, 7-19.

[6] Anderson FE, Williams BW, Horn KM, Erséus C, Halanych KM, Santos SR et al. Phylogenomic analyses of Crassiclitellata support major Northern and Southern Hemisphere clades and a Pangaean origin for earthworms. BMC Evol Biol. 2017; 17(1), 123.

[7] Qiu JP, Bouché M. Révision morphologique, chorologique et taxonomique du genre Scherotheca Bouché, 1972 (Oligochaeta: Lumbricidae). Doc. Pédozool. Integrol. 1998; 4, 117-139.

Formatted: Spanish (Spain)

[8] Qiu JP, Bouché M. Une nouvelle évaluation du genere Helodrilus (sensu Zicsi, 1985) (Oligochaeta: Lumbricidae). Doc. Pédozool. Integrol. 1998; 4, 8-16.

[9] Qiu JP, Bouché M. Le genre Allolobophora Eisen, 1874 (Oligochaeta Lumbricidae), ses avatars et sa définition moderne. Doc. Pédozool. Integrol. 1998; 4, 86-97.

[9] Qiu JP, Bouché M. Une nouvelle évaluation du genere Helodrilus (sensu Zicsi, 1985) (Oligochaeta: Lumbricidae). Doc. Pédozool. Integrol. 1998; 4, 8-16.

[10] Pérez-Losada M., Ricoy M, Marshall JC, Domínguez J. Phylogenetic assessment of the earthworm *Aporrectodea caliginosa* species complex (Oligochaeta: Lumbricidae) based on mitochondrial and nuclear DNA sequences. Mol Phylogenetics Evol. 2009; 52 (2), 293-302.

[11] Pérez-Losada M, Breinholt JW, Aira M, Domínguez J. An updated multilocus phylogeny of the Lumbricidae (Annelida: Clitellata: Oligochaeta) earthworms. J Phylogenetics Evol Biol. 2015; 3 (1).

[12] Domínguez J, Aira M, Porto PG, Díaz Cosín DJ, Pérez-Losada M. Multigene phylogeny reveals two new isolated and relic earthworm genera (Oligochaeta: Lumbricidae). Zool J Linnean Soc. 2018; 182(2), 258-274.

[13] Pérez-Losada M, Breinholt JW, Porto PG, Aira M, Domínguez J. An earthworm riddle: systematics and phylogeography of the Spanish lumbricid *Postandrilus*. PLoS One. 2011; 6 (11).

[14] Paoletti MG, Blakemore RJ, Csuzdi C, Dorigo L, Dreon AL, Gavinelli F. et al. Barcoding *Eophila crodabepis* sp. nov. (Annelida, Oligochaeta, Lumbricidae), a large stripy earthworm from alpine foothills of northeastern Italy similar to *Eophila tellinii* (Rosa, 1888). PloS One. 2016 ; 11 (3).

Formatted: Spanish (Spain)

Formatted: Spanish (Spain)

Formatted: English (United States)

[15] de Sosa I, Díaz Cosín DJ, Csuzdi C, Paoletti MG, Marchán DF. Placing *Eophila tellinii* (Oligochaeta, Lumbricidae) in a molecular phylogenetic context advances the century-old controversy around the problematic genus. Eur J Soil Biol. 2019; 94.

[16] Katoh K, Standley D.M. MAFFT multiple sequence alignment software version 7: improvements in performance and usability. Mol Biol Evol. 2013; 30 (4), 772-780.

[17] Hall TA. BioEdit: a user-friendly biological sequence alignment editor and analysis program for Windows 95/98/NT. Nucleic Acids Symposium Series. 1999; 41, 95-98.

[18] Darriba D, Taboada GL, Doallo R, Posada D. jModelTest 2: more models, new heuristics and parallel computing. Nat Methods. 2012; 9 (8), 772.

[19] Akaike H. Maximum likelihood identification of Gaussian autoregressive moving average models. Biometrika. 1973; 60 (2), 255-265.

[20] Schwarz G. Estimating the dimension of a model. Ann Stat. 1978; 6 (2), 461-464.

[21] Stamatakis A. RAxML Version 8: A tool for Phylogenetic Analysis and Post-Analysis of Large Phylogenies. Bioinformatics. 2014; 30 (9), 1312-1313.

[22] Miller MA., Pfeiffer W, Schwartz T. Creating the CIPRES Science Gateway for inference of large phylogenetic trees. Gateway Computing Environments Workshop (GCE), New Orleans, LA. 2010, pp. 1-8

[23] Ronquist F, Huelsenbeck JP. MrBayes 3: Bayesian phylogenetic inference under mixed models. Bioinformatics. 2003; 19 (12), 1572-1574.

[24] Marchán DF, Fernández R, de Sosa I, Sánchez N, Díaz Cosín DJ, Novo M. Integrative systematic revision of a Mediterranean earthworm family: Hormogastridae (Annelida, Oligochaeta). Invertebr Syst. 2018; 32 (3), 652-671.

[25] Kumar S, Stecher G, Li M, Knyaz C, Tamura K. MEGA X: molecular evolutionary genetics analysis across computing platforms. Mol Biol Evol. 2018; 35 (6), 1547-1549.

[26] Bouché MB. Lombriciens de France: écologie et systématique (Vol. 72, No. 2). Paris: Institut National de la Recherche Agronomique. 1972

[27] Szederjesi T, Pop VV, Pavlícek, Márton O, Krízsik V, Csuzdi C. Integrated taxonomy reveals multiple species in the Dendrobaena byblica (Rosa, 1893) complex (Oligochaeta: Lumbricidae). Zool J Linnean Soc. 2018; 182(3), 500-516.

[28] Cresko WA, Amores A, Wilson C, Murphy J, Currey M, Phillips P. et al. Parallel genetic basis for repeated evolution of armor loss in Alaskan threespine stickleback populations. PNAS. 2004; 101(16), 6050-6055.

[29] Szederjesi T, Angyal D, Balázs G, Dány, L. Remarks on the earthworm genus Helodrilus Hoffmeister, 1845 with new epigean and subterranean records (Oligochaeta, Lumbricidae). Opusc Zool Budapest. 2014; 45(2), 181-188.

[30] Csuzdi, C. Earthworm species, a searchable database. Opusc Zool Budapest. 2012; 43(1): 97-99

Formatted: Default Paragraph Font, Font: (Default) +Body (Calibri), 11 pt, Spanish (Spain)

Supporting information captions

S1. Species included in the reference dataset

S2. Phylogenetic tree obtained by Bayesian phylogenetic analysis of the concatenated sequence of molecular markers *COI*–16S-tRNAs–*ND1*–28S.

S3. Uncorrected average pairwise (UAPG) distances for the marker 16S between representatives of different well-established genera of Lumbricidae.

Reviewer #1: Authors present a very interesting paper about the identity of several Lumbricidae species and subspecies that are placed in a new genus. These new arrangements are due to the use of an integrative approach (molecular genetics, phylogenetic analysis and morphological revisions). English is ok, as well as molecular and phylogenetic methods and results.

We want to thank the reviewer for his/her interest on our manuscript and his/her constructive suggestions in order to improve it. All of them have been considered and included.

There are, however, some modifications that should be done (mainly in regard to classical nomenclatural arrangements) before the paper could be accepted. The paper, thus, is ACCEPTED WITH MINOR CHANGES.

I directly wrote several comments and suggestions over the main text; following are some of my main points:

1) In the title it is mentioned the word Crassiclitellata; in the classification of the new genus Gatesona, however, this word is not included. Thus, include in the supraorder classification this word with its hierarchical associated level.

We have included Crassiclitellata in the taxonomy section.

2) There are several mistakes in regard of the “types” of the two re-described species. Please attend comments of the text.

We have corrected the taxonomic errors regarding the types.

3) As authors do not reviewed types of these species, maybe the name (from an already described species) in fact does not correspond to the collected and molecular sequenced material. I strongly suggest contact Dr. Bouché in order to obtain in loan the types (or paratypes) to confirm that the morphology of Gatesona chaetophora rutena and Gatesona chaetophora lablacherensis really correspond to the species you collected. Even more because there is some variation that could be an indication of different morphological species (e.g. the number of spermathecae).

We have contacted Marcel Bouché and revised the holotypes for *lablacherensis*, *rutena* but also *serninensis* and *chaetophora*. Thanks to that, we can state with certainty that the (morpho-molecularly) studied

material is morphologically compatible with the types of the original taxa. The morphological differences initially detected could be actually due to faulty/incomplete descriptions (in the end, it was done through tables and not through an orthodox text); when comparing with the types, the differences were non-existant or comprised within intra-individual variation (Gatesona rutena holotype possessed three spermathecae on one side but only two on the other).

4) In benefit of readers, please explain in the discussion why the genus was named as Gatesona and not Acystodrilus.

We have included a sentence explaining that point in the "Remarks" section of the Systematic changes to the genus Gatesona.

Reviewer #2: There are a few small corrections of grammar and content in the MS that I uploaded with this review. The revision of the taxonomy has to be modified to make it clear that G. chaetophora is the type species, and in particular, that G. chaetophora chaetophora is the type species. The subspecies G. c. argentatus could be tentatively excluded. The remaining species are "other species" under a heading.

We are thankful for the constructive revision and suggestions for improvement of our manuscript. As seen in the document with tracked changes, we have aknowledged and included all the suggested changes.

Most importantly, we contacted Marcel Bouché and revised the holotypes for *lablacherensis*, *rutena* but also *serninensis* and *chaetophora*. This allowed to check for morphological differences, actually ruling out most of them (holotypes were more consistent with our material tan suggested in the incomplete, informal descriptions provided in Bouché 1972). In addition, we sampled additional material of *Gatesona serninensis*, which allowed us to provide a formal redescription.

We have decided to mantain *argentatensis* within *Gatesona chaetophora* as it clearly belongs to the genus but we lack enough information to robustly elevate it to its own species.
