# Supplementary material for: Systematic revision of Gatesona (Crassiclitellata, Lumbricidae), an endemic earthworm genus from the Massif Central (France)
Source: PLoS One. 2021 Sep 2;16(9):e0255978. doi: 10.1371/journal.pone.0255978 (PMC8412367; doi:10.1371/journal.pone.0255978)
Supplement: Attachment — Submitted filename: Response to reviewers.docx [file pone.0255978.s006.docx]

Reviewer #1: Authors present a very interesting paper about the identity of several Lumbricidae species and subspecies that are placed in a new genus. These new arrangements are due to the use of an integrative approach (molecular genetics, phylogenetic analysis and morphological revisions). English is ok, as well as molecular and phylogenetic methods and results.

**We want to thank the reviewer for his/her interest on our manuscript and his/her constructive suggestions in order to improve it. All of them have been considered and included.**

There are, however, some modifications that should be done (mainly in regard to classical nomenclatural arrangements) before the paper could be accepted. The paper, thus, is ACCEPTED WITH MINOR CHANGES.

I directly wrote several comments and suggestions over the main text; following are some of my main points:

1) In the title it is mentioned the word Crassiclitellata; in the classification of the new genus Gatesona, however, this word is not included. Thus, include in the supraorder classification this word with its hierarchical associated level.

**We have included Crassiclitellata in the taxonomy section.**

2) There are several mistakes in regard of the “types” of the two re-described species. Please attend comments of the text.

**We have corrected the taxonomic errors regarding the types.**

3) As authors do not reviewed types of these species, maybe the name (from an already described species) in fact does not correspond to the collected and molecular sequenced material. I strongly suggest contact Dr. Bouché in order to obtain in loan the types (or paratypes) to confirm that the morphology of Gatesona chaetophora rutena and Gatesona chaetophora lablacherensis really correspond to the species you collected. Even more because there is some variation that could be an indication of different morphological species (e.g. the number of spermathecae).

**We have contacted Marcel Bouché and revised the holotypes for *lablacherensis*, *rutena* but also *serninensis* and *chaetophora*. Thanks to that, we can state with certainty that the (morpho-molecularly) studied material is morphologically compatible with the types of the original taxa. The morphological differences initially detected could be actually due to faulty/incomplete descriptions (in the end, it was done through tables and not through an orthodox text); when comparing with the types, the differences were non-existant or comprised within intra-individual variation (Gatesona rutena holotype possessed three spermathecae on one side but only two on the other).**

4) In benefit of readers, please explain in the discussion why the genus was named as Gatesona and not Acystodrilus.

**We have included a sentence explaining that point in the “Remarks” section of the Systematic changes to the genus Gatesona.**

Reviewer #2: There are a few small corrections of grammar and content in the MS that I uploaded with this review. The revision of the taxonomy has to be modified to make it clear that G. chaetophora is the type species, and in particular, that G. chaetophora chaetophora is the type species. The subspecies G. c. argentatus could be tentatively excluded. The remaining species are "other species" under a heading.

**We are thankful for the constructive revision and suggestions for improvement of our manuscript. As seen in the document with tracked changes, we have aknowledged and included all the suggested changes.**

**Most importantly, we contacted Marcel Bouché and revised the holotypes for *lablacherensis*, *rutena* but also *serninensis* and *chaetophora*. This allowed to check for morphological differences, actually ruling out most of them (holotypes were more consistent with our material tan suggested in the incomplete, informal descriptions provided in Bouché 1972). In addition, we sampled additional material of *Gatesona serninensis*, which allowed us to provide a formal redescription.**

**We have decided to mantain *argentatensis* within *Gatesona chaetophora* as it clearly belongs to the genus but we lack enough information to robustly elevate it to its own species.**
