# Supplementary material for: Systematic revision of Gatesona (Crassiclitellata, Lumbricidae), an endemic earthworm genus from the Massif Central (France)
Source: PLoS One. 2021 Sep 2;16(9):e0255978. doi: 10.1371/journal.pone.0255978 (PMC8412367; doi:10.1371/journal.pone.0255978)
Supplement: Attachment — Submitted filename: Response to reviewers.docx [file pone.0255978.s008.docx]

Reviewer #1: The paper is now ready for its publication. I'm, however, sending in the 2nd round reviewed paper very few suggestions and changes (mainly of style) to improve the paper. These suggestions include change of a word, italics, improve of figure 3 legend, a doubt about the identity of a personal communication and (and this is up to the author to include or not) the addition of a nomenclatural comment.

Thanks for the suggestions. All the changes have been incorporated.
